# Biomimetic Iridium‐Based Photothermal Nanozyme to Trigger Ferroptosis and Pyroptosis and Activate the cGAS‐STING Pathway for Improved Tumor Immunotherapy

**DOI:** 10.1002/advs.202519186

**Published:** 2026-01-20

**Authors:** Lijun Ding, Zhongxiong Fan, Guoyu Xia, Fukai Zhu, Nan Yang, Shujie Yu, Longlong Yuan, Jinyao Li

**Affiliations:** ^1^ School of Pharmaceutical Sciences Institute of Materia Medica Xinjiang University Urumqi China; ^2^ Xinjiang Key Laboratory of Biological Resources and Genetic Engineering College of Life Science and Technology Xinjiang University Urumqi China

**Keywords:** cGAS‐STING pathway, Ferroptosis, Metal‐based nanozyme, Photothermal therapy, Pyroptosis, Tumor immunotherapy

## Abstract

Although nanozymes are potential tumor therapeutics due to their ability to disrupt intracellular redox homeostasis, developing nanozymes with higher therapeutic efficacy and clarifying their antitumor mechanism are challenging. Here, an iridium (Ir)‐based nanozyme (IIN) was constructed through coordination‐driven co‐assembly using photosensitizer indocyanine green (ICG), Ir, and indoleamine 2,3‐dioxygenase (IDO) inhibitor NLG8189. Then, the IIN was mimicked by tumor cell lysate (TCL)‐simulated dendritic cell (DC) membrane to form IIN@M. Based on superior enzyme‐like activity and photothermal performance, IIN@M disrupted the intracellular redox homeostasis by generating reactive oxygen species (ROS) and depleting glutathione (GSH). GSH depletion induced ferroptosis, and ROS burst under photothermal irradiation triggered pyroptosis, thus synergistically enhancing immunogenic cell death (ICD). The generated ROS could promote mitochondrial DNA (mtDNA) oxidative damage and release, finally activating the immune response by the cyclic GMP‐AMP synthase‐simulator of interferon gene (cGAS‐STING) pathway. In vivo experiments also suggested that IIN@M could efficiently ablate the primary tumor, especially under photothermal irradiation. Furthermore, it could suppress distant tumor progression by triggering the immune response, especially under photothermal irradiation, which was accompanied by increased DC maturation, M1 macrophage polarization, and T cell infiltration in tumor tissue. This study proposed a promising strategy for effective Ir‐based nanozyme in tumor immunotherapy.

## Graphical abstract

This study develops an iridium‐based nanozyme (IIN@M) with superior enzyme‐like activity and photothermal performance. It disrupts intracellular redox balance by generating reactive oxygen species and depleting glutathione, which synergistically induces ferroptosis, pyroptosis, and immunogenic cell death. Meanwhile, it activates the Stimulator of Interferon Genes pathway to initiate immune responses. Therefore, it effectively ablates primary tumors and inhibits distant tumor growth.

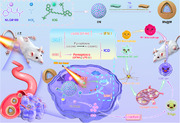

## 1 Introduction

The tumor microenvironment (TME) exhibits several abnormal physiological features, including acidity, hypoxia, and a high level of H_2_O_2_/GSH, due to rapid tumor cell proliferation, abnormal metabolism, and blood supply [1, 2]. These abnormal physiological features provide new insights for developing TME‐responsive antitumor agents [3]. As the nanomaterials with intrinsic enzyme‐like activity, nanozymes can catalyze endogenous substances within TME and trigger tumor catalysis therapy in situ. For instance, they can deplete GSH and catalyze H_2_O_2_ to generate ROS, thereby disrupting the intracellular redox homeostasis and inducing tumor cell death [4]. Due to the good catalytic efficiency, stability, and economy, several types of nanozymes, like peroxidase (POD), catalase (CAT), superoxide dismutase (SOD), glutathione oxidase (GSHOx)‐like nanozymes, have been widely developed and applied in clinical medicine [5, 6].

Due to the multiplex enzyme‐like activities of metal elements, the nanozymes in which metal elements serve as the active site have attracted extensive attention [7]. For example, zinc, copper, cisplatin, ruthenium, and iron‐based metal nanozymes have been developed for tumor therapy [1, 8, 9, 10, 11]. Among numerous metal elements, Ir exhibits well‐catalytic properties due to its multivalence, good adsorption ability to organic compounds, and high physicochemical stability [12, 13]. Ir‐based nanozymes have exhibited positive effects in tumor immunotherapy [14, 15, 16]. For instance, Yao et al. reported that the Ir‐based nanozymes could alleviate hypoxic TME due to their CAT‐like activity, thereby reducing the proportion of CSCs in TME and inhibiting tumor metastasis [17]. Additionally, Wu et al. demonstrated that Ir‐based nanozymes could promote ROS generation in the TME due to their POD‐like activity, thereby promoting tumor ablation through ROS‐mediated apoptotic pathways [18]. Although metal‐based nanozymes show excellent promise in tumor therapy, they only realize limited therapeutic efficacy. Therefore, it is essential to rationally design nanozymes thereby combining various therapeutic approaches to induce multiple forms of cell death. For example, it can be combined with photothermal therapy (PTT) mediated by photosensitizers. PTT has been widely used in tumor therapy and has several advantages, like high selectivity, minimal invasiveness, and strong operability [19]. After absorption of near‐infrared (NIR) light, photosensitizers locally convert light into hyperthermia to ablate tumor cells. It is reported that PTT may help augment blood flow, improve the uptake of chemotherapeutic drugs in the tumor tissues, and even alleviate tumor hypoxia [20]. PTT can directly perform thermal ablation of tumors by inducing pyroptosis and immunogenic cell death (ICD) [21, 22, 23]. The ROS burst during PPT can also promote oxidative damage and release of mtDNA, thereby activating the cGAS‐STING pathway and inducing interferon (IFN) signaling, which is important for enhancing antitumor immune response [6, 24, 25]. It is also reported that the temperature rise during PTT could accelerate the catalytic reaction rate of nanozymes [26, 27]. It is noted that metal elements, such as Ir, can accelerate the intersystem crossing process to generate more ROS during PTT owing to their high spin‐orbit coupling constant. Additionally, it can relieve the aggregation‐caused quenching effects owing to its electronic configuration, thus enhancing the stability and photothermal conversion efficiency of photosensitizers [28]. The high photothermal conversion capabilities are important for PTT [20]. Furthermore, the nanozymes can also be combined with immunomodulators, such as the immune checkpoint inhibitor, STING agonist, and IDO inhibitor, thereby reversing the immune‐suppressive TME [8, 9]. Moreover, cell membrane‐biomimetic techniques, especially derived from the immune cell membrane, have gradually emerged in drug delivery [29]. Cell membrane‐coated nanozymes integrate the natural proteins and molecules on the cell membrane, which play an important role in intercellular crosstalk and endow the targeting to disease sites [30]. Given the essential role of dendritic cells (DCs) in anti‐tumor response, DC membrane‐coated nanoparticles have been widely used in tumor immunotherapy. It is reported that DC membrane‐coated nanoparticles can prolong the circulation time of nanoparticles, enabling their escape from clearance by the immune system, and enhance their tumor targeting, thus inhibiting tumor growth and inducing memory T cell activation for long‐term protective immunity [31, 32, 33, 34].

**SCHEME 1:**
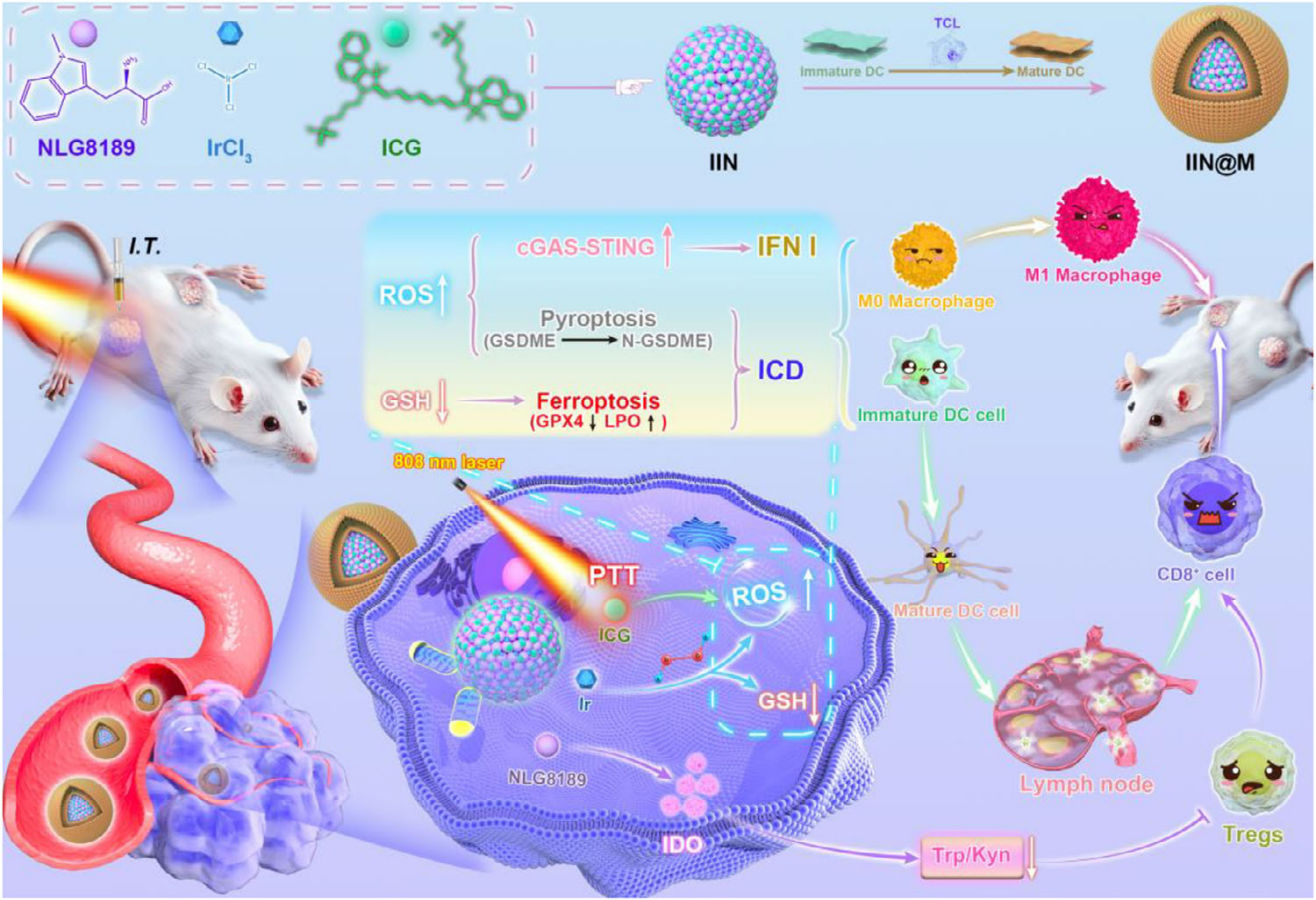
The chematic illustration for the fabrication process of IIN@M nanozyme and its role in enhancing tumor immunotherapy. (1) Depleting GSH and inhibiting GPX4, thus inducing ferroptosis. (2) Causing a ROS burst during PTT, thus inducing pyroptosis. (3) Ferroptosis, pyroptosis, and photothermal effect synergistically enhance ICD. (4) ROS causes mtDNA damage and release, thus activating the cGAS‐STING pathway and immune response.

Here, a multifunctional nanozyme IIN@M was constructed for photothermally improved tumor catalysis therapy and immunotherapy (Scheme 1). The IIN@M was prepared through coordination‐driven co‐assembly using ICG, Ir, and NLG8189, and coated with TCL‐simulated DC membrane. IIN@M owns photothermal activity and triple enzyme‐like activity, including POD, GSHOx, and CAT‐like activity. The GSH depletion could induce ferroptosis, and the ROS burst in the presence of 808 nm laser irradiation could trigger pyroptosis, thus enhancing ICD. Additionally, the generated ROS could promote the damage and release of mtDNA, finally activating antitumor immune response by the cGAS‐STING pathway. These approaches could effectively kill the tumor and trigger the immune response.

## 2 Result and Discussion

### 2.1 Construction and Characterization

**FIGURE 1:**
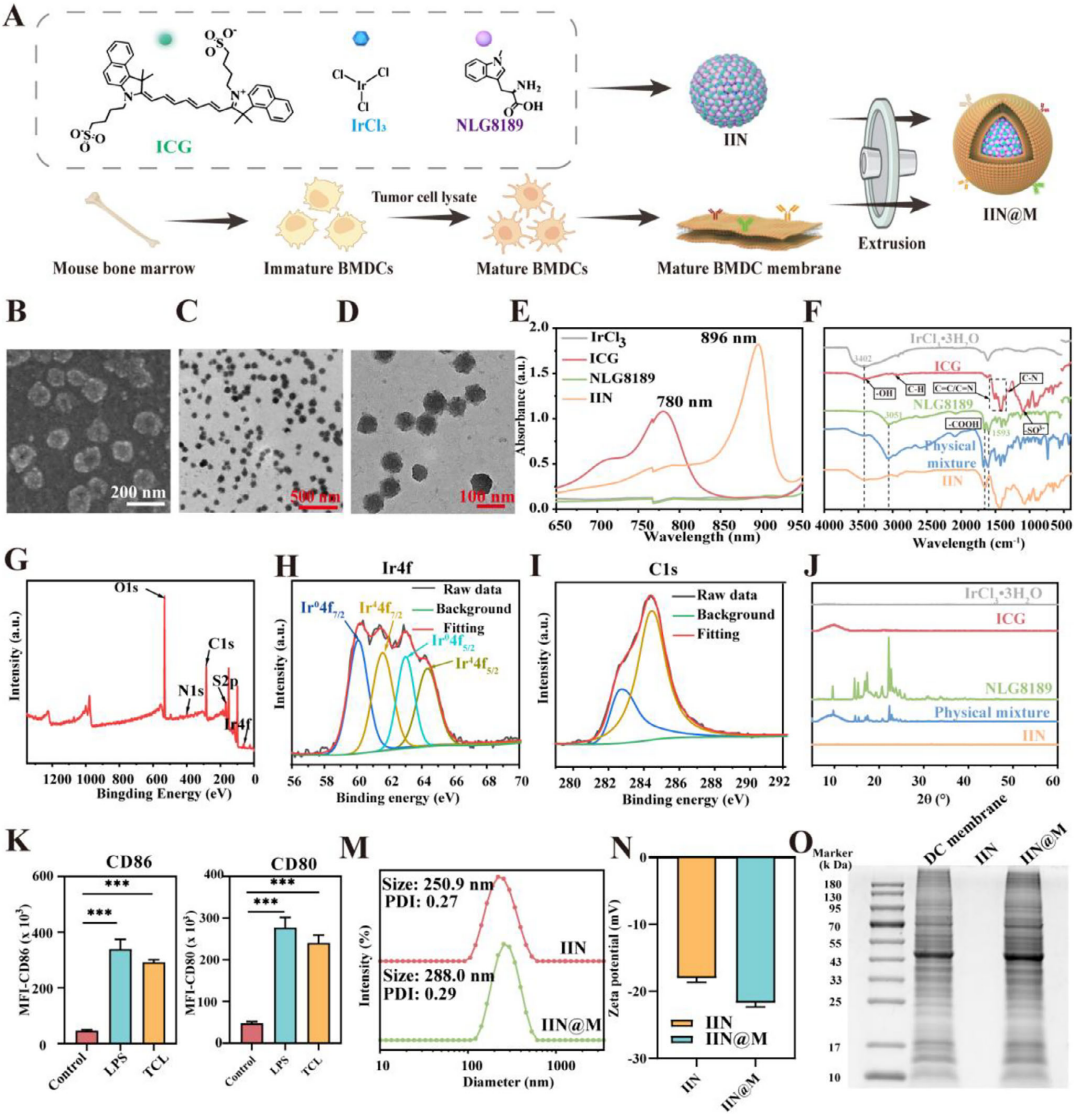
Construction and characterization. (A) Schematic illustration for the synthesis of IIN and IIN@M. (B) Representative SEM image of IIN (scale bar = 200 nm). Representative TEM image of IIN (scale bar = 500 or 100 nm) (C and D). (E) UV–vis spectrum of IrCl_3_, ICG, NLG8189, and IIN solution. (F) FTIR spectrum of IrCl_3_•3 H_2_O, ICG, NLG8189, physical mixture, and IIN. (G‐I) XPS spectrum of IrCl_3_•3 H_2_O, ICG, NLG8189, physical mixture, and IIN. (J) XRD patterns of IrCl_3_•3 H_2_O, ICG, NLG8189, physical mixture, and IIN. (K) The expression level of CD86 and CD80 of DCs after being stimulated by TCL. The Diameter (M) and zeta potential (N) of IIN and IIN@M. (O) SDS‐PAGE analysis of the total proteins from the TCL‐treated DC membrane, IIN, and IIN@M. Figure 1A was created with Biorender.com. Data are expressed as mean ± SD (*n* = 3). ^*^ *p* < 0.05; ^**^ *p* < 0.01; ^***^ *p* < 0.001.

Recently, metal ion‐based coordination‐driven co‐assembly has aroused great interest in formulating delivery systems [30, 35]. Considering the superior enzyme‐like activity and coordination ability of iridium [28], ICG‐Ir‐NLG8189 enzymes (termed as IIN) were constructed through coordination‐driven co‐assembly using small‐molecule photosensitizer ICG, transition metal ion Ir, and small‐molecule IDO inhibitor NLG8189 (Figure 1A). Scanning Electron Microscopy (SEM) image (Figure 1B) and Transmission Electron Microscopy (TEM) (Figure 1C,D) images both revealed that IIN exhibited regularly spherical morphology, uniform size, and excellent dispersion. According to the UV–vis spectrum in Figure 1E, IrCl_3_ solution and NLG8189 solution had no characteristic absorbance peak from 650 to 950 nm. The free ICG solution showed a characteristic absorbance peak at 780 nm due to its unique conjugated system and intramolecular charge transfer characteristics. Notably, the absorbance peak of ICG in IIN displayed a notable red shift to 896 nm, as compared with free ICG. This may be attributed to the fact that ICG, Ir ion, and NLG8189 formed a supramolecular coordination structure with a metal‐to‐ligand charge transfer feature. This coordination structure significantly extended the conjugated system and enhanced the charge transfer capacity of ICG, thereby reducing the energy required for electron transition from the ground state to the excited state, and finally resulting in a red shift of the absorbance peak from 780 to 896 nm. To further investigate the coordination mechanism of IIN, the Fourier Transform Infrared (FTIR) spectrum was obtained. In the FTIR spectrum (Figure 1F), the peak shape of IIN changed compared with that of IrCl_3_•3H_2_O, ICG, NLG8189, and their physical mixture, indicating that chemical bond interaction occurred in IIN. In the FTIR spectrum of IIN, the characteristic peak at 1420 cm^−1^ assigned to C═C/C═N stretching vibration of ICG became broader, and that of ‐SO_3_^−^ vibration at 1087 cm^−1^ became weaker. Additionally, the characteristic peak at 1593 cm^−1^ assigned to the C═O stretching vibration of NLG8189 disappeared in IIN. These changes in characteristic peaks suggested that these functional groups are involved in the coordination interaction with Ir, such as the formation of Ir‐N and Ir‐O coordination bonds. Moreover, the O‐H stretching vibration of H_2_O in the IIN spectrum shifted to 3393 cm^−1^ and became weaker compared with that of IrCl_3_•3H_2_O, indicating that the crystal water was replaced during the coordination formation, and the formation of coordination bonds altered the hydrogen bonding network and molecular vibrational mode.

The X‐ray Photoelectron Spectroscopy (XPS) and X‐ray diffraction (XRD) patterns were performed to identify the chemical state and surface properties of IIN. XPS spectrum revealed the presence of C, N, O, S, and Ir elements (Figure 1G). High‐resolution spectra in the Ir 4f region (Figure 1H) suggested that the Ir in IIN was present in two chemical states: Ir (0) and Ir (4). Ir (0) was characterized by 4f_7/2_ and 4f_5/2_ with binding energies of 60.08 and 62.98 eV, and Ir (4) was characterized by 4f_7/2_ and 4f_5/2_ with binding energies of 61.58 and 64.28 eV, respectively, which is beneficial to the enzyme‐like catalytic performance [36]. As shown in Figure 1J, the XRD pattern of IrCl_3_•3 H_2_O was relatively flat with no obvious sharp diffraction peak. ICG had an extensive amorphous diffraction peak, and NLG8189 had the sharp crystalline diffraction peaks. However, the sharp crystalline diffraction peaks of NLG8189 disappeared in IIN, indicating that IIN existed in an amorphous state. This may be because the well‐organized crystal lattice was gradually disrupted as the coordination reaction proceeded due to the nano‐scale effect of IIN.

To realize the biomimetic modification to obtain the IIN@M, TCL‐simulated DC membrane was mixed with IIN and physically extruded using polycarbonate films with pore sizes of 800 and 450 nm. After being treated with TCL for 24 h, the expression level of CD80 and CD86 on DCs was significantly increased compared with the control group (Figure 1K), suggesting the DCs were mature. Then, the hydrodynamic size and zeta potential of IIN and IIN@M were measured. The diameter of IIN was 250.9 nm with a polydispersity index (PDI) of 0.27, and the diameter of IIN@M was increased to 288.0 nm with a polydispersity index (PDI) of 0.29, due to surface coating with TCL‐treated DC membrane (Figure 1M). According to Figure 1N, the zeta potential of IIN was −18.03 mV, due to the effective conjugation of negatively charged ICG [37]. After being coated with TCL‐treated DC membrane, the zeta potential of IIN@M was decreased to −21.72 mV. The results of the diameter and zeta potential both suggested successful membrane coating. To further prove the successful membrane coating, the Sodium dodecyl sulphate‐polyacrylamide gel electrophoresis (SDS‐PAGE) was performed to compare the total proteins of TCL‐treated DC membrane, IIN, and IIN@M. Results showed no obvious difference between the protein bands of TCL‐treated DC membrane and IIN@M, revealing that IIN@M maintained the integrity of TCL‐treated DC membrane protein during the coating process. Additionally, the TEM image of IIN@M also suggested the successful coating of TCL‐activated mature DC membrane on IIN (Figure S2). To directly quantify the membrane coating efficiency, the percentage of IIN successfully coated with TCL‐activated mature DC membrane was evaluated using the DiO probe by a flow cytometer. DiO is a general dye for cellular membranes with green fluorescence. Before incorporation into the cellular membrane, DiO exhibits very weak fluorescence. However, after embedding into the membrane, it shows green fluorescence, and the fluorescence intensity significantly increases. The DiO fluorescence intensity of both uncoated IIN and TCL‐activated mature DC membrane‐coated IIN@M was measured using flow cytometry. The percentage of DiO‐positive particles corresponds to the proportion of IIN successfully coated with TCL‐activated mature DC membrane. As shown in Figure S1, the percentage of the DiO‐positive population in the IIN group was 28.1%, which may be because of non‐specific adsorption or a small amount of DiO molecules. For the IIN@M group, the DiO‐positive population was obviously increased to 87.9%. This result suggested that approximately 60% of the IIN was successfully coated with the TCL‐activated mature DC membrane. The successful coating of the TCL‐activated mature DC membrane could further enhance the tumor targeting and anti‐tumor effects of IIN.

The cumulative release curves of ICG, IIN, and IIN@M under simulated physiological PBS (pH 7.4) are shown in Figure S3. The free ICG group exhibited rapid release within the first 8 h, stabilizing after 12 h with a cumulative release of approximately 60%. This result demonstrated the typical “burst release” effect, characterized by an initial rapid drug release that makes it difficult to maintain long‐term therapeutic concentrations and leads to swift clearance in vivo. In contrast, the IIN group shows sustained and gradual release kinetics. Due to encapsulation by DC cell membranes, IIN@M exhibits an even more prolonged and controlled release profile, achieving a cumulative release of approximately 30% after 72 h. This sustained‐release property of IIN@M is beneficial for prolonging its circulation time in vivo, thereby enhancing its delivery efficiency to tumor tissues and improving its therapeutic efficacy.

### 2.2 Enzyme‐Like Catalytic Activity

**FIGURE 2:**
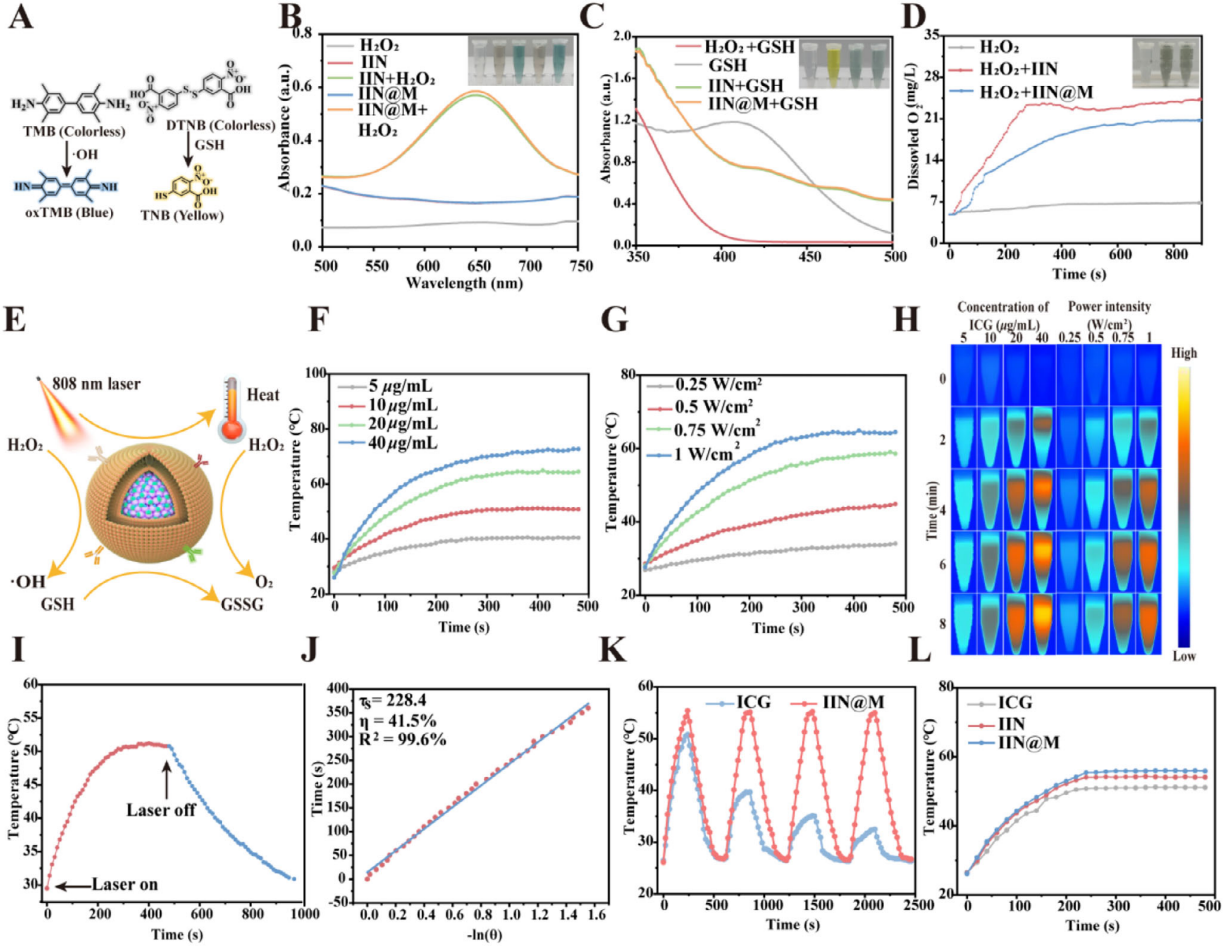
Enzyme‐like activity and photothermal performance in vitro. (A) The probe and reaction mechanism used to detect POD and GSHOx‐like activity. (B) The absorption spectra of •OH generation detection using TMB probe. (C) The absorption spectra of GSH depletion detection using the DTNB probe. (D) The dissolved oxygen curves with different treatments. (E) The schematic illustration of the enzyme‐like activity and photothermal performance of IIN@M. Photothermal photos (H) and rise curves of IIN@M at different concentrations (F) and power intensity (G). Temperature rise curve during heating and cooling (I) and photothermal conversion efficiency of IIN@M (J). (K) Recycling‐heating profiles of IIN@M for 4 lase on/off cycles. (L) Photothermal rise curves of ICG, IIN, and IIN@M at the same concentration and power intensity.

It has been confirmed that Ir‐containing nanodrugs possess several enzyme‐like catalytic activities [36]. The peroxidase (POD)‐like activity of IIN and IIN@M was examined using the 3,3’,5,5’‐tetramethylbenzidine (TMB) probe. The colorless TMB can be oxidized by •OH to form blue ox‐TMB. Compared with H_2_O_2_, IIN, and IIN@M group, a significant increase of absorbance at 562 nm was observed in IIN+ H_2_O_2_ and IIN@M+ H_2_O_2_ group, and the solution of IIN+ H_2_O_2_ and IIN@M+ H_2_O_2_ group became blue (Figure 2B). This indicates that IIN and IIN@M own good POD‐like activity, which allows them to catalyze H_2_O_2_ to form •OH within the TME, thus inhibiting the tumor. Additionally, we evaluated the effect of different concentrations of H_2_O_2_ (6.25, 12.5, 25, 50, 100 mm) on the POD reaction rate, with a reaction time of 5 min. Using the Michaelis‐Menten equation fitting, the K_m_ and V_max_ of the POD‐mimicking activity were 1.664 × 10^−5^ M/min and 44.03 mm, respectively (Figure S5).

The glutathione oxidase (GSHOx)‐like activity of IIN and IIN@M was assessed using the 5,5’‐dithiobis (2‐nitrobenzoic acid) (DTNB) probe. Colorless DTNB can form yellow 5‐thio‐2‐nitrobenzoic acid (TNB) in the presence of GSH, and TNB has an absorbance peak at 412 nm. Compared with the GSH group, the absorbance at 412 nm of the IIN/IIN@M+GSH group was decreased, revealing the good GSHOx‐like activity of IIN and IIN@M (Figure 2C). The abundant endogenous GSH within tumor cells can scavenge the generated ROS, thus diminishing the chemo‐dynamic effect [12]. However, the glutathione oxidase (GSHOx)‐like activity of IIN and IIN@M enables them to deplete GSH and enhance chemodynamic therapy effect. Additionally, the ability of IIN and IIN@M to deplete GSH can induce tumor cell ferroptosis.

The catalase (CAT)‐like activity of IIN and IIN@M was evaluated by monitoring the generation of O_2_ by a portable dissolved oxygen meter. As depicted in Figure 2D, the O_2_ concentration in the IIN/IIN@M group reached as high as approximately 20 mg/mL after reaction with H_2_O_2_ for 15 min, reflecting that both IIN and IIN@M possessed good CAT‐like activity and can efficiently catalyze H_2_O_2_ to form O_2_. The generation of O_2_ can efficiently alleviate the hypoxic state of TME, promote tumor vascular normalization, and inhibit tumor cell metastasis [38]. Notably, the O_2_ production capability of the IIN@M group was slightly lower than that of the IIN group, which may be attributed to the protective effect of the DC membrane, which hindered the complete reaction of Ir with H_2_O_2_, thereby attenuating O_2_ production.

### 2.3 Photothermal Performance

We evaluated the photothermal performance of IIN@M under 808 nm laser irradiation. According to the thermal images and temperature curves under different concentrations and power intensities shown in Figure 2F,G, the IIN@M had an excellent photothermal performance, and its temperature change exhibited concentration and power intensity dependence. The photothermal conversion efficiency (η) of IIN@M was calculated to be 41.5% according to Roper's method, which was superior to that of free ICG, commonly reported range of 16%–34% [39, 40, 41, 42, 43, 44]. This result indicates that IIN@M has an excellent photothermal conversion performance. Additionally, IIN@M showed higher photothermal stability than free ICG (Figure 2K). After 4 heating‐cooling cycles, the IIN@M almost keeps the same photothermal performance, while free ICG showed photothermal instability. The excellent photothermal stability of IIN@M may be attributed to its stable coordinated nanostructure. Additionally, Figure 2L showed that the photothermal performance of IIN@M was superior to that of IIN owing to the protective effect of the DC membrane. Collectively, the coordinated nanostructure endowed the excellent photothermal property to IIN@M and made it a promising photothermal agent for tumor therapy.

Upon 808 nm laser irradiation, ICG transferred energy to O_2_, thus generating oxidizing singlet oxygen (^1^O_2_) The ability of IIN and IIN@M to generate ^1^O_2_ under 808 nm laser irradiation was evaluated using the DPBF probe. DPBF can be specifically oxidized by ^1^O_2_, resulting in a decrease of its characteristic absorbance at 410 nm. As shown in Figure S4, the absorbance of DPBF at about 410 nm obviously decreased in the ICG+L group compared to the control group, indicating the ^1^O_2_ generation of ICG upon 808 nm laser irradiation. Interestingly, the absorbance was further decreased in IIN+L and IIN@M+L groups compared with the ICG+L group, which confirmed that nanozyme effectively generated ^1^O_2_.

### 2.4 Cellular Internalization and Cytotoxicity In Vitro

**FIGURE 3:**
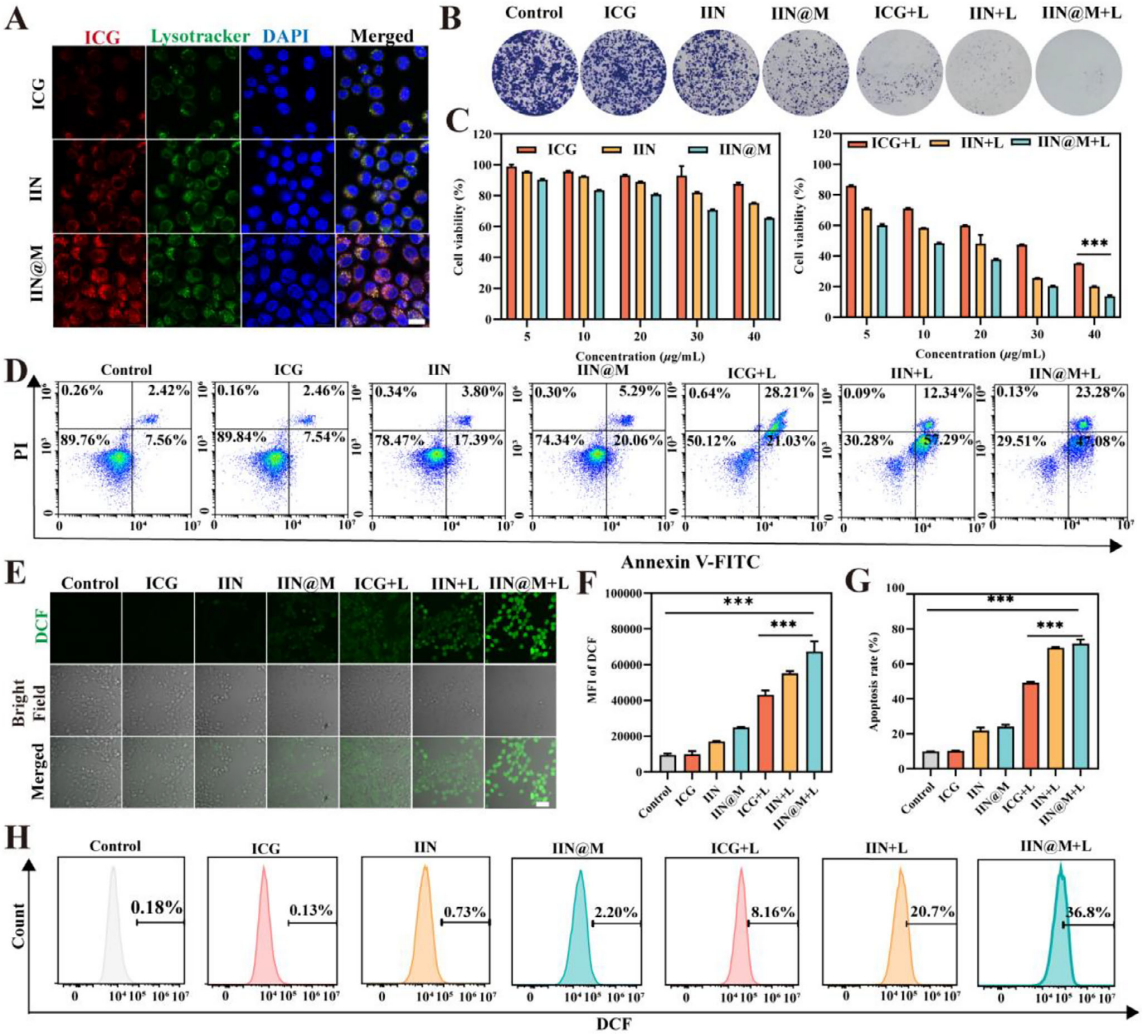
Cellular internalization and cytotoxicity in vitro. (A) CLSM image of subcellular localization of ICG, IIN, and IIN@M after incubation with 4T1 cells for 8 h (scale bar = 20 *µm*). (B) Clone formation assay of 4T1 cells after being treated with ICG, IIN, and IIN@M with or without 808 nm laser irradiation. (C) Cell viability of 4T1 cells after being treated with ICG, IIN, and IIN@M with or without 808 nm laser irradiation. Apoptosis (D) and percentage of apoptotic 4T1 cells (G) with different treatments. Detection of intracellular ROS generation using DCFH‐DA probe by CLSM (E) and flow cytometry (F and H) (scale bar = 50 *µm*). ICG+L, IIN+L, and IIN@M+L refer to the group treated with ICG, IIN, and IIN@M plus 808 nm laser irradiation, respectively. Data are expressed as mean ± SD (*n* = 3). ^*^ *p* < 0.05; ^**^ *p* < 0.01; ^***^ *p* < 0.001.

The internalization efficiency by tumor cells is essential for the antitumor effects. The cell uptake of ICG, IIN, and IIN@M was monitored by a flow cytometer, after co‐culturing with 4T1 cells for 2, 4, and 8 h. A higher intracellular ICG fluorescence intensity indicates a higher internalization efficiency of ICG/IIN/IIN@M. As shown in Figure S7, the fluorescence intensity increased with time, indicating that ICG/IIN/IIN@M all exhibited time‐dependent cell internalization over the studied time periods. At the same time, the higher fluorescence intensity of IIN and IIN@M indicated their own higher internalization efficiency than free ICG, which may be attributed to their nanostructure with rough surface and larger specific surface area. It is noted that IIN@M had the highest internalization efficiency, which may be because the DC membrane coating increased the receptor‐mediated endocytosis. The CLSM analysis obtained similar results (Figure S6). The detailed internalization mechanism of IIN and IIN@M was elucidated using Lysotracker by CLSM. According to Figure 3A, after incubation with IIN and IIN@M, a substantial amount of red fluorescence was observed in lysosomes, which is significantly higher than that of free ICG. These results suggested that IIN and IIN@M entered lysosomes after being taken up by 4T1 cells, which is consistent with the research reported previously [45]. The acidic environment within lysosomes can promote the disruption and release of nanodrugs.

The MTT cytotoxicity of ICG, IIN, and IIN@M to 4T1 with or without 808 nm irradiation was investigated. The cell viability of 4T1 after different treatments was decreased with a significant concentration dependence. When the concentration was 40* µ*g/mL, the cell viability of the IIN and IIN@M group was decreased to 75.3% and 65.6% (Figure 3C), respectively, indicating their antitumor effects. After 808 nm irradiation with 1 W/cm^2^ for 5 min, ICG/IIN/IIN@M+L groups all exhibited lower cell viability. The IIN@M+L group showed the lowest cell viability (13.2%), which may be attributed to its higher internalization efficiency and superior induced effect of ferroptosis, pyroptosis, and ICD, ultimately causing synergistic killing of tumor cells. According to the results of the clone formation assay, compared with the control and ICG group, IIN and IIN@M could inhibit the growth and proliferation of 4T1 cells, and the photothermal effect could further promote the inhibitory effects (Figure 3B). Moreover, the apoptosis assay was further conducted to evaluate the antitumor effect of IIN and IIN@M. Similarly, after 808 nm laser irradiation, 4T1 cells showed a higher apoptosis rate (Figure 3D,G). The apoptosis rate of the IIN@M+L group was highest and was as high as 71.6%.

Additionally, MTT cytotoxicity assay of IIN@M toward normal HEK293 cells was performed. As shown in Figure S8, even at the highest tested concentration of 40 *µ*g/mL, the cell viability of the IIN@M group was still as high as 86%, indicating that IIN@M showed a relatively lower cytotoxicity against HEK293 cells. At the same concentration of IIN@M, the cell viability of 4T1 was only 65%. These phenomena can be attributed to a unique TME with lower pH, higher concentrations of H_2_O_2_ and GSH. Compared to normal cells, tumor cells exhibit a lower pH, higher concentrations of H_2_O_2_ and GSH. The concentration of H_2_O_2_ in tumor cells is approximately 100‐fold higher than that in normal cells, while that of GSH is about 4‐10‐fold [46, 47]. Under the acidic tumor microenvironment, IIN@M can catalyze H_2_O_2_ to generate highly cytotoxic •OH due to their POD‐like activity, causing tumor cell death. Additionally, it can deplete GSH due to its GSHOx‐like activity, thereby disrupting the intracellular redox balance and promoting ferroptosis.

### 2.5 ROS Burst‐Induced Pyroptosis In Vitro

The disruption of intracellular redox homeostasis initiated by ROS accumulation is a vital factor for inducing tumor cell death [30]. The ROS burst, especially triggered by PTT, can activate caspase 3, and the generated cleaved caspase 3 can cleave GSDME, thereby inducing pyroptosis [22]. Extracellular ROS level was determined using 2’,7’‐Dichlorodihydrofluorescein diacetate (DCFH‐DA) probe, which can be oxidized by intracellular ROS and form 2’,7’‐Dichlorodihydrofluorescein with green fluorescence. Compared with the control and ICG group, the IIN/IIN@M group showed weak green fluorescence (Figure 3E). Once treated by 808 nm laser irradiation, 4T1 cells showed brighter green fluorescence, indicating photothermal effect could promote intracellular ROS generation. Compared with the ICG+L group, IIN/IIN@M+L groups generated more ROS, which may be attributed to the POD‐like activity of IIN/IIN@M. Due to high internalization efficiency, the IIN@M+L group generated more ROS than the IIN+L group. These results were consistent with the results obtained by flow cytometry (Figure 3F,H).

Additionally, as reported previously, the photosensitizer undergoes electron energy level transition under near‐infrared light irradiation, thereby forming the “triplet excited state,” which transfers energy to O_2_ and makes it form highly ^1^O_2_ [20]. Therefore, the intracellular level of ^1^O_2_ after different treatments was evaluated using the SOSG probe by the flow cytometer. The SOSG itself exhibits weak fluorescence, but after specific reaction with ^1^O_2_, it shows a highly green fluorescence. According to Figure S9, the fluorescent intensity was increased after being treated with ICG, IIN, and IIN@M plus 808 nm laser irradiation, indicating the generation of ^1^O_2_. The IIN@M+L group showed the highest level of intracellular ^1^O_2_, which is consistent with its enhanced cellular uptake facilitated by the DC membrane coating, thus inducing a more efficient ability to generate ^1^O_2_.

**FIGURE 4:**
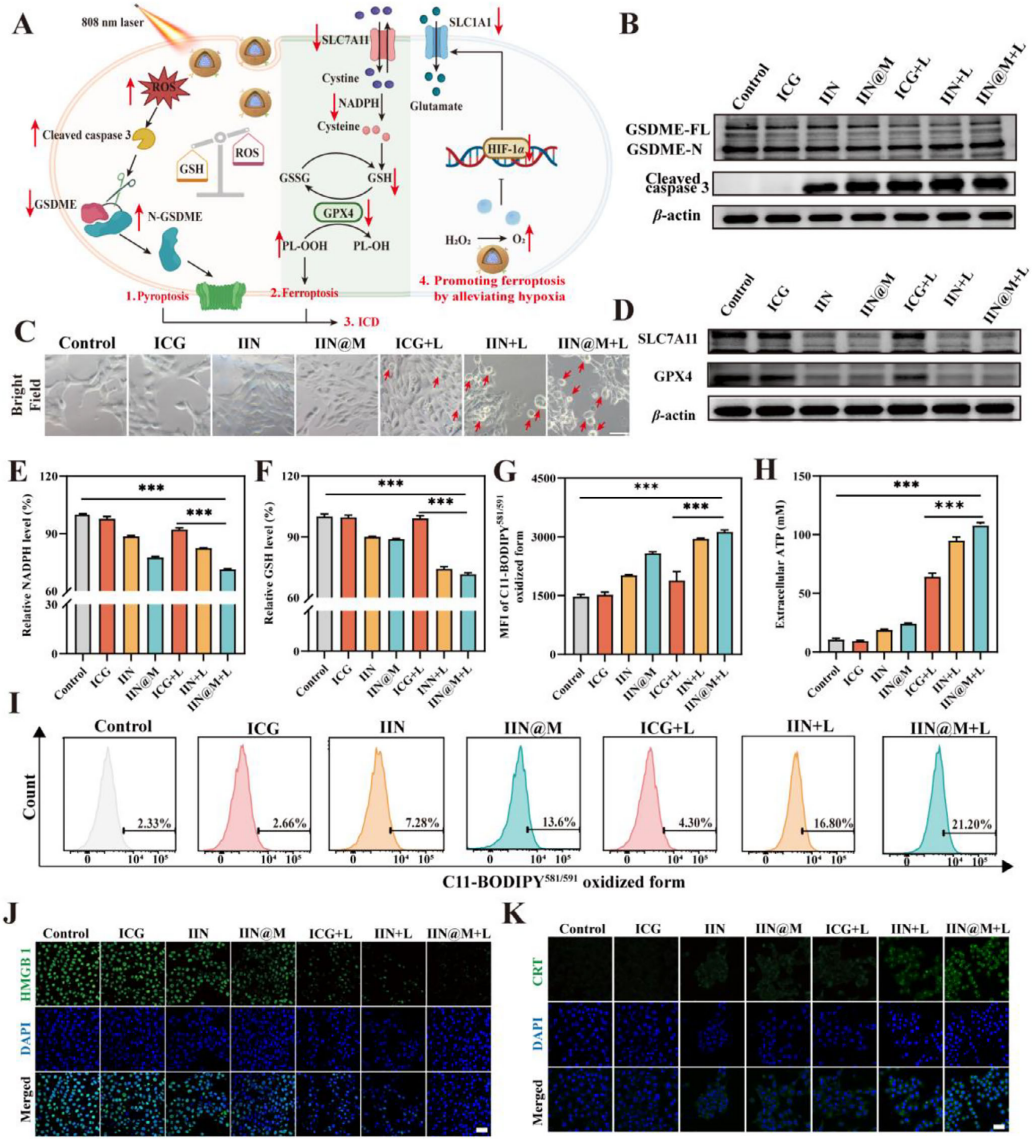
The induction of ferroptosis, pyroptosis, and ICD in vitro. (A) Mechanism diagram of pyroptosis, ferroptosis, and ICD triggered by IIN@M with 808 nm laser irradiation. (B) The expression level of GSDME‐FL, N‐GSDME, and cleaved caspase 3. (C) Pyroptosis images of 4T1 cells with different treatments (scale bar = 20 *µm*). (D) The expression level of SLC7A11 and GPX4. Intracellular NADPH (E) and GSH (F) levels. Representative flow cytometry plots (I) and quantitative analysis (G) of LPO level using C11‐BODIPY^581/591^ probe. The ATP (H) and HMGB1(J) release and CRT exposure (K) (scale bar = 50 *µm*). Data are expressed as mean ± SD (*n* = 3). ^*^ *p* < 0.05; ^**^ *p* < 0.01; ^***^ *p* < 0.001. Figure 4A was created with Biorender.com.

Then, we investigated the ROS burst‐induced pyroptosis by assessing the intracellular expression level of cleaved caspase 3, GSDME precursor (GSDME‐FL), and cleaved GSDME (N‐GSDME) by western blot. As shown in Figure 4B, the expression level of cleaved caspase 3 was increased after treatment with IIN and IIN@M alone, and this can be further increased after treatment with IIN and IIN@M plus 808 nm laser irradiation. This may be attributed to the fact that the burst of ROS under 808 nm irradiation causes mitochondrial dysfunction along with a decrease in mitochondrial membrane potential (MMP) and opening of mitochondrial permeability transition pore (mPTP) [22]. Then, the cleaved caspase 3 cleaved the GSDME. Correspondingly, western blot results showed that after being treated by ICG, IIN, and IIN@M plus 808 nm laser irradiation, the level of GSDME‐FL was decreased, and the GSDME primarily existed in the form of N‐GSDME (Figure 4B). The N‐GSDME polymerizes to form pores on the cell membrane, leading to the release of intracellular LDH. As shown in Figure S11B, the LDH in 4T1 cell supernatant was increased after being treated with ICG/IIN/IIN@M plus 808 nm laser irradiation. Then, the cell morphology was observed using an optical microscope. Results showed that the cells of the group treated without 808 nm laser irradiation exhibited intact and spindle‐shaped, while those of ICG+L, IIN+L, and IIN@M+L exhibited the prominent features, i.e., elliptic and irregular swelling and membrane rupture (indicated by red arrows in Figure 4C), which is consistent with the results reported previously [48]. Moreover, it is reported that the formation of membrane pores and swelling of the cell membrane can also cause the release of ATP, etc. [49]. Indeed, the ATP content in the supernatant of ICG+L, IIN+L, and IIN@M+L groups was significantly increased, and that of IIN@M+L was the most significant (Figure 4H). Taken together, the Ir‐based nanozymes (IIN and IIN@M) could effectively generate ROS under 808 nm laser irradiation, thus inducing pyroptotic cell death by activating the caspase 3/GSDME signal pathway.

### 2.6 GSH Depletion Induced Ferroptosis In Vitro

The ability of metal‐based nanozymes to deplete GSH can trigger ferroptosis. Ferroptosis is programmed cell death characterized by iron‐dependent intracellular accumulation of lipid hydroperoxide (LPO). We verified the ability of IIN and IIN@M to induce cellular ferroptosis by investigating several ferroptosis‐related indicators, including GSH, NADPH, GPX4, LPO, etc. According to Figure 4E, the intracellular relative NADPH level significantly decreased after being treated by IIN and IIN@M with or without 808 nm laser irradiation, compared with the control and ICG group. Similarly, results showed that the intracellular relative GSH level decreased by10.0% and 11.2% after being treated by IIN and IIN@M, compared with the control and ICG group, whereas the relative GSH level after being treated by IIN and IIN@M plus 808 nm laser irradiation decreased by 25.9% and 28.5% (Figure 4F). These results indicated that IIN and IIN@M can deplete intracellular GSH due to the presence of Ir, and this process can be facilitated by photothermal irradiation, which is consistent with previous literature [50]. As a critical regulator of ferroptosis, GPX4 is a GSH‐dependent peroxidase that can affect LPO accumulation, and the depletion of GSH inhibits the activity of GPX4. [12]. We examined the intracellular expression level of GPX4 through western blot and detected the intracellular LPO level by flow cytometer. Results showed that the expression level of GPX4 was downregulated after treatment of IIN and IIN@ with or without 808 nm laser irradiation compared with other groups, which was in correspondence with the decrease of intracellular GSH level (Figure 4F). The results were in accordance with the results reported previously [51]. The decrease in GPX4 level may be due to the intracellular GSH depletion and ROS generation [12, 50, 51]. Following the reduction of GPX4 level, the fluorescent intensity of C11‐BODIPY^581/591^ was significantly increased after treatment of IIN and IIN@M (Figure 4G,I), indicating that IIN and IIN@M caused a significant intracellular accumulation of LPO. The accumulation of LPO was further promoted after being treated by IIN and IIN@M plus 808 nm laser irradiation. This is consistent with the results obtained from CLSM analysis (Figure S11C). As the primary product of lipid peroxidation, the intracellular MDA obviously increased after treatment of IIN and IIN@M, suggesting that they could promote intracellular MDA accumulation. Meanwhile, this could be promoted by photothermal effects (Figure S11A). Taken together, these results suggest that IIN and IIN@M can trigger ferroptosis, and NIR irradiation can further promote ferroptosis. Additionally, it is reported that relieving hypoxia within tumor cells could further promote ferroptosis [52]. The intracellular O_2_ level was detected using [Ru(dpp)_3_] Cl_2_ probe. Its red fluorescence can be quenched by O_2_. Results showed that the fluorescence intensity of the IIN and IIN@M group was decreased, indicating that IIN and IIN@M could relieve the hypoxic state in 4T1 cells (Figure S10A). The generated O_2_ inhibited the expression of hypoxia‐inducible factor 1*α* (HIF‐1*α*), which further inhibited the expression of ferroptosis‐related SLC1A1, thus promoting ferroptosis (Figure S10B)

### 2.7 Synergistically Induced ICD In Vitro

Several studies have reported that PTT, ferroptosis, and pyroptosis can synergistically induce tumor ICD [1, 48, 51, 53]. ICD is a cell death type characterized by exposure and release of damage‐associated molecular pattern (DAMP), including exposure of calreticulin (CRT), secretion of high‐mobility group box 1 (HMGB1) protein, and release of adenosine triphosphate (ATP) [1]. These DAMPs release the “eat‐me” signal, therefore activating the antitumor immune response by simulating DC maturation and M1 macrophage polarization. In this study, the CRT exposure (Figure 4K), HMGB1 secretion (Figure 4J), and ATP release (Figure 4H) of 4T1 cells were measured after various treatments. After being treated by IIN and IIN@M, the 808 nm laser irradiation group had a notable ATP release, HMGB1 migration from nucleus to extracellular area, CRT secretion, and exposure to cell membrane, confirming that PTT could efficiently induce ICD. Additionally, the cells treated by IIN and IIN@M without 808 nm laser irradiation could also induce slight ATP release, HMGB1 migration, and CRT secretion.

### 2.8 ROS‐induced mtDNA Release and Activation of the STING Pathway

**FIGURE 5:**
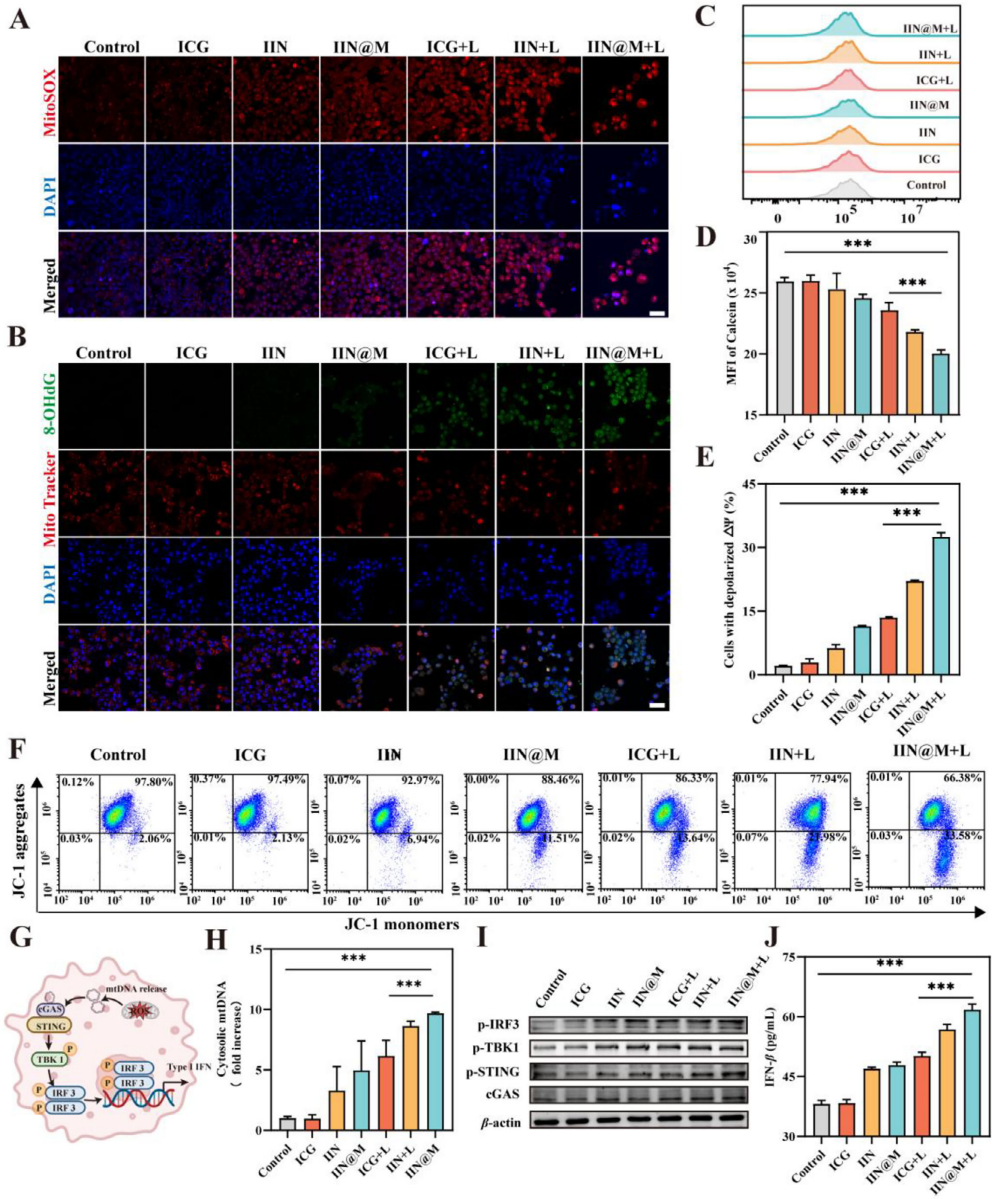
ROS‐induced mtDNA release and activation of the cGAS‐STING pathway. (A) The CLSM images of mitochondrial ROS generation (scale bar = 50 *µm*). (B) The CLSM images of DNA oxidative damage within 4T1 cells (scale bar = 50 *µm*). Representative flow cytometry plots (C) and quantitative analysis (D) of mPTP opening using Calcein‐AM probe. Representative flow cytometry plots (F) and quantitative analysis (E) of MMP changes by JC‐1 staining. (G) Mechanism diagram of the activation of the cGAS‐STING pathway. (H) The cytosolic mtDNA level after different treatments. (I) The expression level of cGAS, p‐STING, p‐TBK1, and p‐IRF3. (J) Enzyme‐linked immunosorbent assay (ELISA) measurement of IFN‐*β* in cocultivation suspension of 4T1 cells with different treatments. Data are expressed as mean ± SD (*n* = 3). ^*^ *p* < 0.05; ^**^ *p* < 0.01; ^***^ *p* < 0.001. Figure 5G was created with Biorender.com.

The generated ROS causes mitochondrial dysfunction, then the ROS‐damaged mtDNA is released into the cytoplasm and activates the cGAS‐STING pathway [54]. The mitochondrial ROS was detected using the MitoSOX probe. MitoSOX can specifically accumulate in mitochondria and is selectively oxidized by mitochondrial superoxide, finally generating red fluorescence. As shown in Figure 5A, compared with the control and ICG group, the IIN/IIN@M group showed a slightly increased red fluorescence, and the ICG/IIN/IIN@M+L group showed higher red fluorescence, indicating that IIN/IIN@M induced the generation of mitochondrial ROS, and this process could be enhanced by the photothermal effect. The generation of mitochondrial ROS was attributed to the damage of oxidative phosphorylation and mitochondrial electron transport chain after being simulated by the drug and photothermal effect [55]. Due to a lack of histone protection, mtDNA has a weak self‐repair ability and is highly susceptible to mitochondrial ROS damage [56]. Then, the oxidative damage of DNA was detected by measuring the level of 8‐hydroxy‐2’‐deoxyguanosine (8‐OHdG), which is the primary biomarker of DNA oxidative damage. The anti‐8‐OHdG antibody was labelled with FITC and showed green fluorescence. As illustrated in Figure 5B, the IIN@M group showed slight green fluorescence within mitochondria, while the ICG/IIN/IIN@M+L group showed higher green fluorescence, indicating stronger mtDNA under 808 nm laser irradiation. According to the staining of DAPI and Mito Tracker, the mtDNA and nuclear DNA were both damaged under 808 nm laser irradiation, and IIN@M+L showed the strongest mtDNA and nuclear DNA oxidative damage.

The mitochondrial membrane permeability is important for the release of mtDNA into the cytoplasm, and it was assessed by measuring the opening of mPTP and changes in MMP. The opening of mPTP was detected using Calcein AM. As illustrated in Figure 5C,D, the MFI of ICG/IIN/IIN@M+L group, especially the IIN@M+L group, was significantly decreased, indicating that the 808 nm laser irradiation triggered the opening of mPTP, thus Calcein was released from mitochondria to cytoplasm, where its fluorescence was quenched by CoCl_2_. Then, the MMP was measured using the JC‐1 probe. JC‐1 is present in aggregate form in normal mitochondria, while it is present in monomer form in damaged mitochondria. According to Figure 5E,F, cells with depolarized *△Ψ* were increased to 6.3% and 11.5% after being treated by IIN and IIN@M, while that of the IIN/IIN@+L group was increased to 22.1% and 32.5%, respectively. This result indicated IIN/IIN@M could slightly decrease MMP, while 808 nm laser irradiation could further promote this process owing to more ROS generation. The decrease of MMP can further promote the opening of mPTP.

Furthermore, the mtDNA release from mitochondria into the cytoplasm was quantified by qPCR. After being treated with IIN/IIN@M, the cytosolic mtDNA exhibited a 3‐fold and 5‐fold increase compared to the control group, while that of IIN/IIN@M+L exhibited 9‐fold and 10‐fold increase, respectively (Figure 5H). This indicated that the ROS generation and mitochondrial membrane permeability increase could enable mtDNA to be released into the cytosol. The mtDNA in the cytosol can activate the cGAS‐STING pathway through direct binding to cGAS. To verify the activation of the cGAS‐STING pathway, a western blot was performed to assess the expression level of STING‐related proteins in 4T1 cells. As illustrated in Figure 5I, IIN and IIN@M not surprisingly increased the expression of cGAS and the phosphorylation level of STING, TBK1, and IRF3. Synergistic photothermal effect further elevated their expression level because of ICG‐mediated photosensitivity, finally promoting the activation of the cGAS‐STING pathway. The activation of cGAS‐STING can simulate the secretion of type I interferon (IFN‐I), therefore initiate adaptive immunity and enhance tumor immunotherapy [57, 58]. As an essential IFN‐I, IFN‐*β* can simulate the maturation and cross‐presentation of DCs, thus promoting T lymphocyte immunity [59]. Then, we detected the level of IFN‐*β* in the supernatant of 4T1 cells with different treatments. Compared with the control and ICG group, IFN‐*β* in the other groups was all increased. Compared with the control group, the IFN‐*β* in the IIN@M group increased 1.6‐fold. Similar results were also observed in that of IL‐6 and TNF‐*α* (Figure S12). Taken together, these results confirmed that IIN/IIN@M and photothermal effect‐triggered mtDNA release could effectively activate the cGAS‐STING pathway and promote the secretion of IFN‐. It is noted that although many studies have confirmed that the cytosolic mtDNA could bind to cGAS to activate the STING pathway [60, 61], the direct co‐localization imaging of cytosolic mtDNA with cGAS should be performed in future studies.

### 2.9 The Immune Activation In Vitro

**FIGURE 6:**
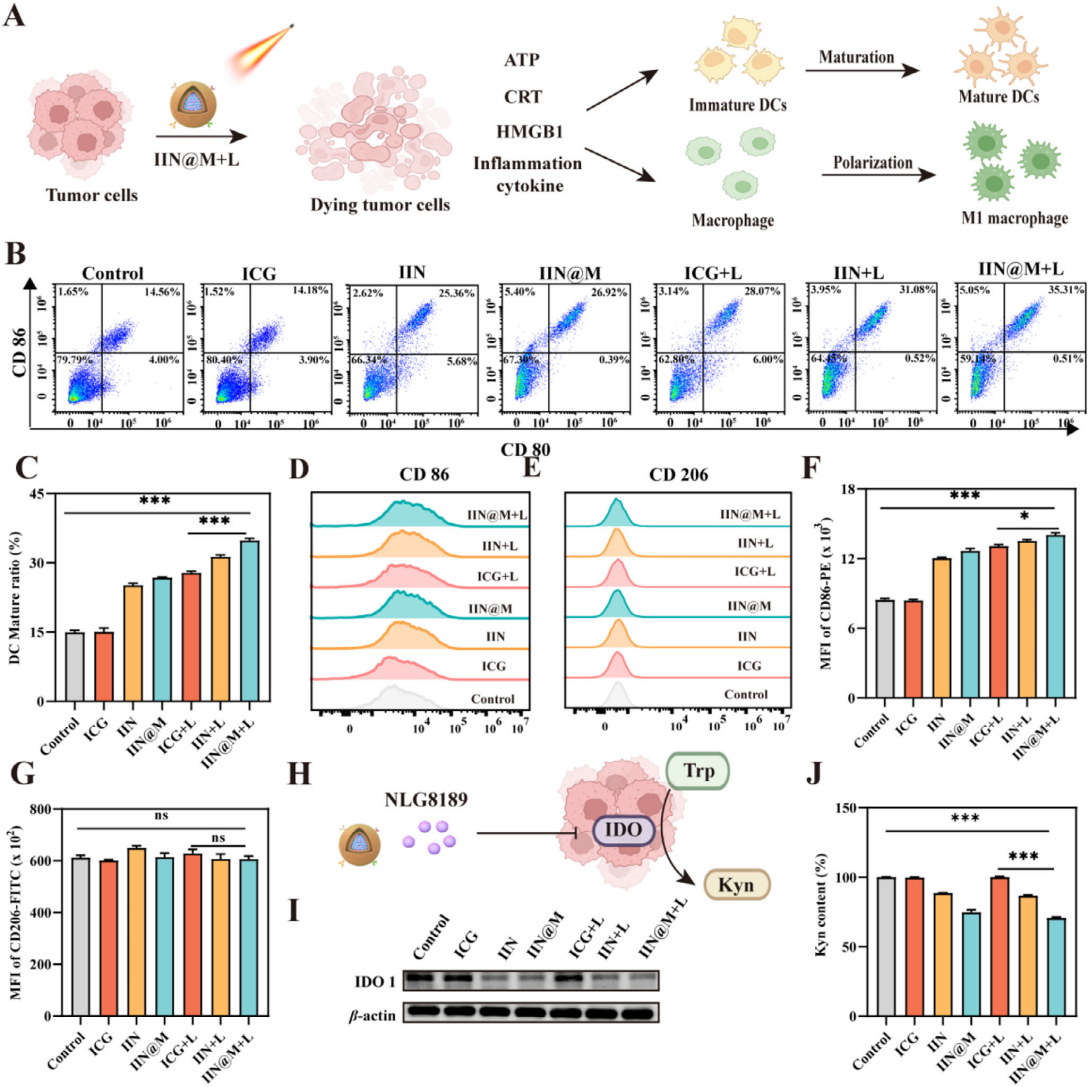
The immune activation and IDO activity inhibition in vitro. (A) The schematic illustration of DC maturation and macrophage polarization. Representative flow cytometry plots (B) and quantitative analysis (C) of DC maturation ratio. Flow cytometric analysis of RAW 264.7 cell polarization to M1 (D and F) and M2 (E and G) type macrophage after co‐incubation with supernatant of 4T1 cells from each treatment group. (H) Schematic illustration of IDO activity inhibition by IIN@M. (I) The expression level of IDO 1 in 4T1 with different treatments. (J) The Kyn content in the 4T1 suspension from each treatment group. Data are expressed as mean ± SD (*n* = 3). ^*^ *p* < 0.05; ^**^ p < 0.01; ^***^ *p* < 0.001. Figure 6A,H were created with Biorender.com.

It is reported that the secretion and release of IFN‐*β*, ATP, HMGB1, and CRT mentioned above can trigger the activation and maturation of DCs and induce the polarization of M1 macrophages [62]. The BMDCs were co‐cultured with supernatants of 4T1 cells with different treatments for 12 h, and the DC mature ratio was evaluated by measuring the maturation markers, CD80 and CD86. As illustrated in Figure 6B,C, compared with the control and ICG group, the ratio of CD86^+^CD80^+^DC in other groups was increased. It is noted that the ratio of CD86^+^CD80^+^DC in IIN/IIN@M+L groups was as high as 31.1% and 35.3%, respectively, indicating a higher DC maturation rate. This is attributed to their more effective induction of ferroptosis, pyroptosis, and ICD as well as activation of the cGAS‐STING pathway, causing increased secretion and release of DAMPs and inflammatory cytokines. To determine their effect on macrophage polarization, the M0 macrophages, RAW 264.7 cells, were co‐cultured with supernatants of 4T1 cells with different treatments for 12 h. Then, the M1 and M2 macrophages were detected by assessing CD86 and CD206, respectively. According to Figure 6E,G, no significant difference in MFI of CD206 was observed among all groups. Compared with the control and ICG group, the MFI of CD86 in other groups was all increased, and the IIN@M+L group showed the highest MFI (Figure 6D,F). These results revealed that IIN/IIN@M could promote macrophages to polarize to M1 macrophages, and IIN@M plus photothermal treatment showed a stronger effect.

### 2.10 IDO Activity Inhibition In Vitro

IDO, highly expressed in breast cancer, plays an essential role in tumor immunosuppression. It can catalyze the tryptophan (Trp) to form kynurenine (Kyn), which can inhibit the proliferation of cytotoxic T lymphocytes and impair their activity, thus promoting immune escape [51]. NLG8189, the component of IIN and IIN@M, is a common IDO1 inhibitor. The effect of IIN/IIN@M on the expression and activity of IDO1 was measured. As depicted in Figure 6I, the intracellular expression level of IDO1 was notably decreased after being treated with IIN/IIN@M with or without 808 nm laser irradiation. Additionally, compared with the control and ICG group, a remarkable decrease in Kyn content was observed in the IIN/IIN@ and IIN/IIN@M+L group (Figure 6J). These results suggested that IIN and IIN@M can effectively inhibit the activity of IDO1 and Kyn generation. It is reported that the downstream metabolites of Kyn, including 3‐kynurenine (3HK) and 3‐hydroxyanthranilic acid (HAA), can scavenge ROS and inhibit ferroptosis [51, 63]. Therefore, the inhibition effect of IIN/IIN@M on Kyn generation could promote tumor cell ferroptosis. Additionally, Kyn is reported to upregulate the expression of cystine/glutamate antiporter SLC7A11, then promote the biosynthesis of glutathione, and finally inhibit ferroptosis [63]. We investigated the expression of SLC7A11 and found that the expression of SLC7A11 was notably decreased after treatment of IIN/IIN@M with or without 808 nm laser irradiation (Figure 4D). Taken together, the IIN and IIN@M can inhibit the activity of IDO, reverse immunosuppression, and enhance the therapeutic effect of ferroptosis.

### 2.11 The Biosafety and Anti‐Tumor Effect In Vivo

**FIGURE 7:**
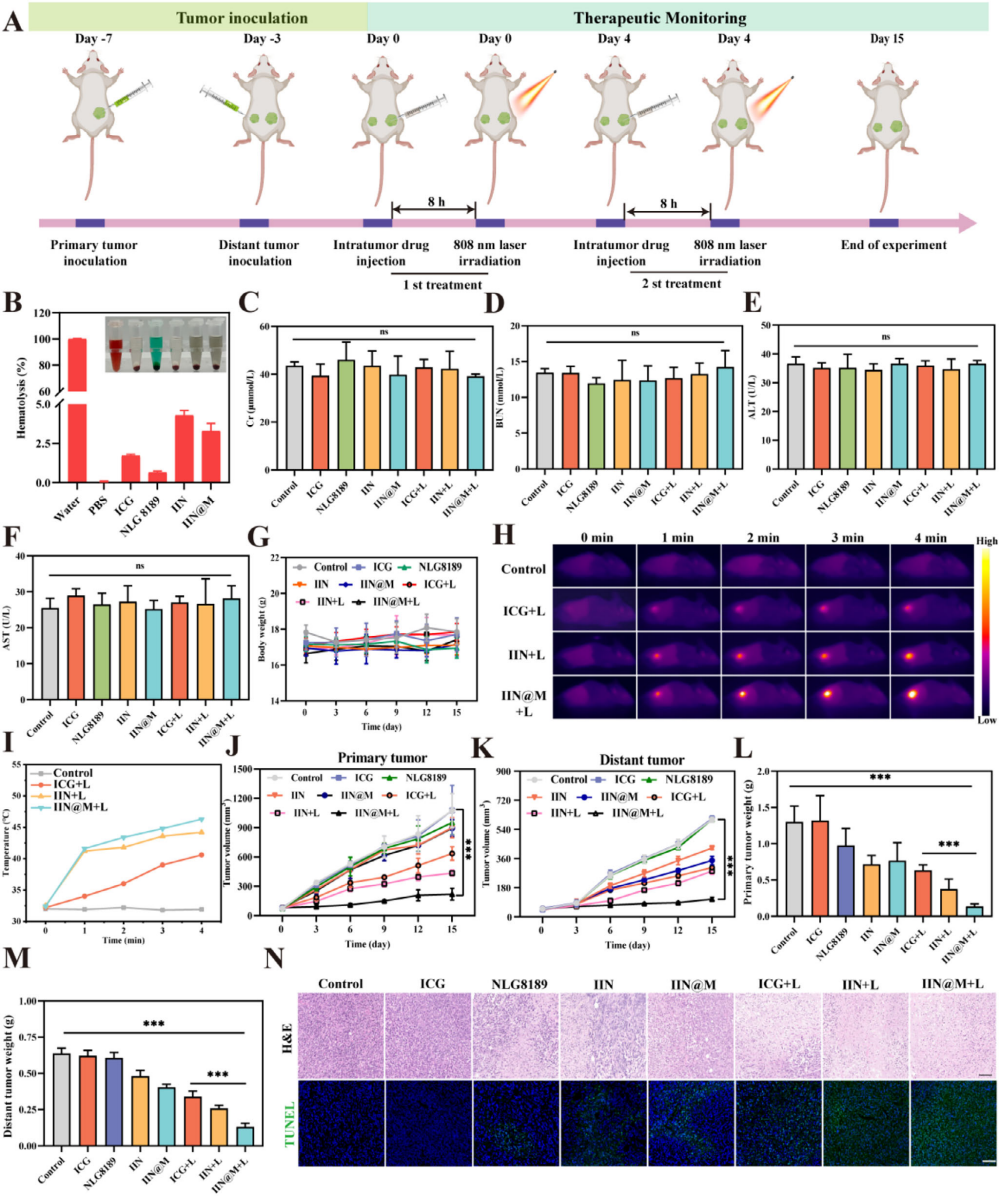
Therapeutic efficacy mediated by IIN@M in vivo. (A) Schematic representation of the treatment schedule. (B) Hemolysis rates of water, PBS, ICG, NLG8189, IIN, and IRD@M. Serum biochemistry parameters, including Cr (C), BUN (D), ALT (E), and AST (F) levels. (G) Body weight changes of mice after treatment. In vivo thermal imaging images (H) and temperature curve (I) of 4T1‐tumor‐bearing mice treated with ICG, IIN, and IIN@M after 8 h injection upon 808 nm laser irradiation. Growth curves of tumor volumes of the primary (J) and distant tumor (K) after different treatments. The weight of the primary (L) and distant (M) tumors after various treatments. Histological microscopic images (N). The dissected primary tumors were stained with H&E and TUNEL (green fluorescence). Scale bar = 100* µm*. Data are expressed as mean ± SD (*n* = 5). ^*^ *p* < 0.05; ^**^ *p* < 0.01; ^***^ *p* < 0.001.

As illustrated in Figure 7A, the anti‐tumor effect was further investigated in a 4T1 tumor model. 4T1 cells were subcutaneously inoculated into both flanks of BABL/C mice, termed as primary tumor and distant tumor, at day −7 and day −3, respectively. When the tumor volume reached approximately 80 mm^3^, the mice were randomly divided into 8 groups: Control, ICG, NLG8189, IIN, IIN@M, ICG+L, IIN+L, and IIN@M+L. On day 0, the drugs were intratumorally injected at the site of primary tumors with a dose of 10 mg/kg. After injection for 8 h, 808 nm laser irradiation was performed in the laser groups (1 W/cm^2^, 4 min). The biosafety in vivo was evaluated because good biosafety is a vital prerequisite for biomedical agents. The hemolysis rates of all drugs (ICG, NLG8189, IIN, and IIN@M) at a concentration of 200 *µg*/mL were all below 5%, suggesting their good hemocompatibility and absence of acute hemolysis risk (Figure 7B). The level of creatinine (Cr) (Figure 7C), urea nitrogen (BUN) (Figure 7D), alanine aminotransferase (ALT) (Figure 7E), and aspartate aminotransferase (AST) (Figure 7F) in the serum of each treatment group showed no significant difference compared with the control group. The body weight of mice in each treatment group showed no significant changes compared with the control group, indicating that all the administration was well tolerated (Figure 7G). Furthermore, the H&E staining images showed that all drugs had no notable tissue injury (Figure S14). These results all suggested that the tested drug had good biocompatibility and biosafety.

Benefiting from the excellent biological properties of the natural cellular membrane, we subsequently investigated the in vivo biodistribution of IIN@M in a 4T1‐1 tumor‐bearing mouse model. After intravenous injection of ICG, IIN, and IIN@M for 8 h, the mice were sacrificed, and their hearts, livers, spleens, lungs, spleens, and tumors were collected for NIR *ex vivo* fluorescence imaging. According to Figure S13, ICG is distributed in the livers, spleens, lungs, kidneys, and tumor tissues, with no specific tumor‐targeting ability. The highest accumulation is observed in the liver, indicating that ICG is primarily metabolized through the hepatic pathway. In the IIN group, the fluorescence intensity of the tumor is higher than that of other tissues, indicating the tumor‐targeting ability of IIN. The tumor‐targeting ability of IIN may be attributed to the Enhanced Permeability and Retention (EPR) effect. Compared with IIN, the IIN@M showed more efficient targeting ability toward the tumor, suggesting that coating with TCL‐stimulated DC membranes further enhances tumor‐targeting specificity, which is also consistent with findings reported previously [32, 64]. This phenomenon may be attributed to the tumor‐associated antigens and homing molecules expressed on its surface.

The photothermal imaging showed that the treatment of IIN@M resulted in a rapid temperature increase to 46.3°C in the tumor site with 4 min of 808 nm laser irradiation (Figure 7H,I). This result indicated that IIN@M is well‐suited for in vivo PTT. During the 15‐day treatment, the tumor volume curves showed that IIN and IIN@M could suppress primary tumor growth, compared with the control group, likely due to their ability to generate ·OH and deplete GSH, leading to enhanced cellular oxidative stress and promotion of cell death (Figure 7J). Meanwhile, the growth of the distant tumor in the IIN and IIN@M groups was also inhibited, which may be attributed to the immune activation after they triggered tumor cell death (Figure 7K). Furthermore, all laser groups showed more significant antitumor effects, and the IIN@M + L group showed the most significant antitumor effects. Tumor weight (Figure 7L,M) and *ex vivo* tumor imaging (Figure S15) were consistent with trends in tumor volume changes, highlighting a pronounced antitumor effect of IIN@M + L. The antitumor efficacy was further evaluated through the terminal deoxynucleotidyl transferase‐mediated deoxyuridine triphosphate nick end labeling (TUNEL) staining assay and hematoxylin and eosin (H&E) staining assay. Consistent with the tumor suppression results, the tumor in the IIN@M plus 808 nm laser irradiation group showed the strongest green fluorescence in TUNEL staining images, revealing the massive tumor cell apoptosis after treatment (Figure 7N). Similar results of tumor tissue damage were also obtained from H&E staining (Figure 7N).

### 2.12 Activation of the Immune Response In Vivo

**FIGURE 8:**
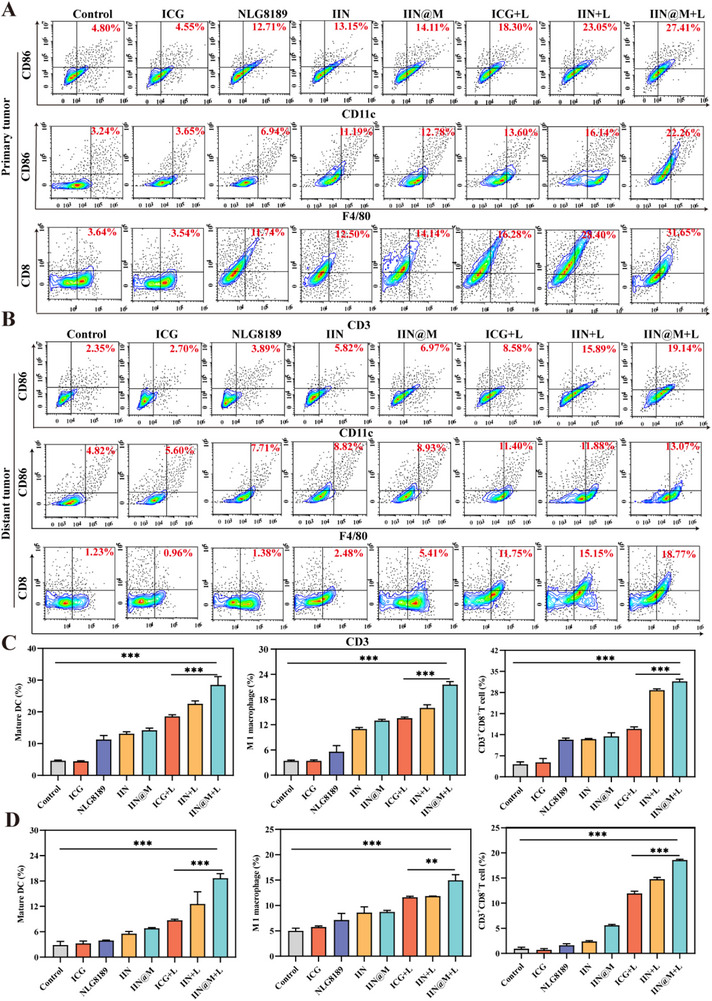
Activation of the immune response in vivo. Flow cytometric analysis of DC maturation (CD86^+^CD11c^+^), M1 macrophage polarization (F4/80^+^CD86^+^), and T cell infiltration (CD3^+^CD8^+^) in primary (A) and distant tumor (B). Quantitative analysis of DC maturation (CD86^+^CD11c^+^), M1 macrophage polarization (F4/80^+^CD86^+^), and T cell infiltration (CD3^+^CD8^+^) in primary (C) and distant tumor (D). Data are expressed as mean ± SD (*n* = 3). ^*^ *p* < 0.05; ^**^ *p* < 0.01; ^***^ *p* < 0.001.

As the professional antigen‐presenting cell, DCs play a vital role in the activation and differentiation of CD8^+^ cytotoxic T lymphocytes that are essential in the anti‐immune response. Additionally, as one of the most abundant immune cells in the TME, macrophages also play a crucial role in tumor growth, invasion, metastasis, and immunosuppression. Therefore, the infiltration of immune cells, including mature DCs (CD86^+^CD11c^+^), M1 macrophage polarization (F4/80^+^CD86^+^), and T cell infiltration (CD3^+^CD8^+^) into primary and distant tumor tissues was investigated using Flow cytometry (Figure 8). It is found that the mature DCs, M1 macrophage, and CD8^+^T cells were all increased in both primary and distant tumors, and the infiltration of these immune cells was moderately further increased in the 808 nm laser irradiation groups, especially in the IIN@M+L group. Results showed that IIN@M+L increased the infiltrated mature DCs ratio to 27.41% and 19.14% in primary and distant tumors, respectively, and that of CD8^+^T cells was as high as 31.65% and 18.77%. Similarly, the infiltrated M1 macrophages were increased to 22.26% and 13.07% in primary and distant tumors, respectively. Taken together, the IIN/IIN@M+L could boost DCs maturation, M1 macrophage polarization, and CD8^+^T‐cells infiltration, thus promoting the elimination of tumor cells.

## 3 Conclusion

In summary, we successfully developed an iridium‐based photothermal nanozyme coated with TCL‐activated DC membrane (IIN@M). Based on its superior enzyme‐like activity and photothermal performance, it could disrupt the intracellular redox homeostasis by generating ROS and depleting GSH, thus inducing tumor cell death and immune activation. The GSH depletion could decrease the activity of GPX4, thereby inducing ferroptosis, and the ROS burst in the presence of 808 nm laser irradiation could trigger pyroptosis. Ferroptosis, pyroptosis, and photothermal effect synergistically enhance ICD. Additionally, the generated ROS could promote the damage and release of mtDNA, finally activating antitumor immune response by the cGAS‐STING pathway. In vivo experiments also suggested that IIN@M could efficiently ablate the primary tumor, especially through PTT mediated by ICG. Furthermore, it could suppress the progression of distant tumors by triggering the immune response, especially in the presence of the 808 nm laser irradiation, which was accompanied by enhanced DC maturation, M1 macrophage polarization, and increased CD8^+^ T cell infiltration within the tumor tissue.

## Author Contributions

L. D. did Conceptualization, Writing – original draft, and Data curation. Z. F. did Conceptualization, Supervision, and Funding acquisition. G. X. did Data curation. F. Z. did Data curation. N. Y. did Data curation. S. Y. did Data curation. L. Y. did Data curation. J. L. did Conceptualization, Supervision, and Funding acquisition.

## Ethics Approval and Consent to Participate

All animal experiments were conducted in accordance with the guidelines of the Ethics Committee of Xinjiang University (XJUAE‐2025‐011).

## Conflicts of Interest

The authors declare no conflicts of interest.

## Supporting information




**Supporting File**: advs73759‐sup‐0001‐SuppMat.docx.

## Data Availability

Research data are not shared.
