# Supplementary material for: Biomimetic Iridium‐Based Photothermal Nanozyme to Trigger Ferroptosis and Pyroptosis and Activate the cGAS‐STING Pathway for Improved Tumor Immunotherapy
Source: Adv Sci (Weinh). 2026 Jan 20;13(16):e19186. doi: 10.1002/advs.202519186 (PMC13042847; doi:10.1002/advs.202519186)
Supplement: Supplementary file 1 — Supporting File: advs73759‐sup‐0001‐SuppMat.docx. [file ADVS-13-e19186-s001.docx]

***Supplementary Material***

**Biomimetic iridium-based photothermal nanozyme to trigger ferroptosis and pyroptosis and activate the cGAS-STING pathway for improved tumor immunotherapy**

Lijun Ding ^a, b^, Zhongxiong Fan*^a^, Guoyu Xia ^a, b^, Fukai Zhu ^a, b^, Nan Yang ^a, b^, Shujie Yu ^a, b^, Longlong Yuan ^a, b^, Jinyao Li ^*b^

^a^ School of Pharmaceutical Sciences, Institute of Materia Medica, Xinjiang University, Urumqi 830017, China

^b^ Xinjiang Key Laboratory of Biological Resources and Genetic Engineering, College of Life Science and Technology, Xinjiang University, Urumqi 830017, China

^*^ Corresponding authors: Zhongxiong Fan: E-mail: [fanzhongxiong@xju.edu.cn](mailto:fanzhongxiong@xju.edu.cn);

Jinyao Li: E-mail: ljyxju@xju.edu.cn;

**1 Experimental section**

**1.1 Materials**

Indocyanine green (ICG, 95% purity), Iridium (III) chloride trihydrate (IrCl_3_•3H_2_O, 98% purity), 3,3',5,5'-tetramethylbenzidine (TMB, 98% purity), hydrogen peroxide solution (H_2_O_2_, 3 wt% in water), 30% trichloroacetic acid solution (w/v), and p-dimethylaminobenzaldehyde/acetic acid was from Shanghai Macklin Biochemical Co., Ltd. (Shanghai, China). 3-(4,5-Dimethylthiazol-2-yl)-2,5-diphenyltetrazolium bromide (MTT, 98% purity), 1,3-diphenylisobenzofuran (DPBF, ≥97% purity), and JC-1 probe were purchased from Aladdin Biological Technology Co., Ltd. (Shanghai, China). [Ru(dpp)_3_] Cl_2_ was obtained from Maokang Biotechnology Co., Ltd (Shanghai, China). Indoximod (NLG8189, 97% purity) was obtained from Merck KGaA Co., Ltd. (Darmstadt, Germany). Murine recombinant granulocyte/macrophage colony-stimulating factor (GM-CSF) was obtained from Thermo Fisher Scientific (MA, USA). Glutathione reduced (GSH) and 5,5'-dithiobis (2-nitrobenzoic acid) (DTNB, 98% purity) were both purchased from Yuanye Biology Co., Ltd. (Shanghai, China). DiO (DiOC_18_(3)) probe was purchased from Yeasen Biotechnology (Shanghai) Co., Ltd. (Shanghai, China). 2′,7′-Dichlorodihydrofluorescein diacetate (DCFH-DA), Singlet Oxygen Assay Kit with SOSG, Hoechst 33342, MitoSOX Red probe, Mito tracker, mitochondria isolation buffer, radioimmunoprecipitation assay (RIPA) buffer, inhibitors of proteases and phosphatases, anti-GPX4 antibody, anti-IDO1 antibody, anti-HIF-1*α* antibody, and anti-p-TBK1 antibody were obtained from Beyotime Biotechnology Co., Ltd. (Shanghai, China). Anti-8-OHdG antibody was obtained from Solarbio Science & Technology Co., Ltd. (Beijing, China). Anti-CD80 antibody, anti-CD86 antibody, anti-CD11c antibody, anti-CD11b antibody, anti-F4/80 antibody, anti-CD206 antibody, anti-CD3 antibody, anti-CD4 antibody, and anti-CD8 antibody were all obtained from Elabscience Biotechnology Co., Ltd. (Wuhan, China). Anti-CRT antibody was purchased from Santa Cruz Biotechnology Co., Ltd (Shanghai, China). Anti-HMGB1 antibody and FITC-labelled anti-rabbit IgG (H+L) antibody were purchased from Aibotec Biotechnology Co. Ltd. (Wuhan, China). Anti-*β*-actin antibody was obtained from Servicebio Co., Ltd. (Wuhan, China). Anti-cGAS antibody, anti-p-STING antibody, and anti-p-IRF3 antibody were all purchased from HUABIO Co., Ltd (Hangzhou, China). Anti-cleaved caspase 3 antibody, anti-GSDME antibody, anti-SLC7A11 antibody, and anti-SLC1A1 antibody were all purchased from Proteintech Co., Ltd (Wuhan, China).

**1.2 Synthesis of IIN**

To begin with, 1 mL of ICG (2 mg/mL) aqueous solution and 1 mL of NLG8189 (0.4 mg/mL) methanol solution were slowly added to 2 mL of IrCl_3_•3H_2_O (1.4 mg/mL) aqueous solution, respectively. Next, the mixture was stirred (500 rpm) at room temperature for about 12 h in the dark. Then, the methanol was removed by vacuum-evaporating using a rotary evaporator (CCA-1112A, Shanghai Ailang, China) at 37 ℃. Finally, the crude product was washed by three centrifugations (10,000 rpm, 15 min) to obtain IIN.

**1.3 Characterization of IIN**

The hydrodynamic diameter, polydispersity (PDI), and zeta potential were measured by a Zetasizer (Nano ZS90, Malvern Instruments, UK). The morphology was observed using a Transmission Electron Microscopy (TEM, JEM-2010, JEOL, Japan) and a Scanning Electron Microscopy (SEM, SU8010, HITACHI, Japan). The UV-Vis spectra were recorded using a spectrophotometer (UV2355, Unico, China). The Fourier Transform Infrared (FTIR) spectra were obtained using a FTIR spectrometer (VERTEX 70, BRUKER, Germany). X-ray Diffraction (XRD) analysis was carried out on an X-ray Diffractometer (D8 Advance, BRUKER, Germany). X-ray Photoelectron Spectroscopy (XPS) was measured using an X-ray Photoelectron Spectrometer (ESCAlab250 xi, Thermo Scientific, America).

**1.4 Extraction of tumor cell lysate (TCL)-activated dendritic cell (DC) membrane and construction of IIN@M**

The preparation of TCL was carried out as reported previously with some modifications [1, 2]. Briefly, 4T1 cells were cultured with RPMI-1640 medium containing 10% fetal bovine serum and 1% penicillin-streptomycin at 37 °C in 5% CO_2_ atmosphere. Next, the 4T1 cells were collected and lysed in hypotonic lysis buffer for 1 h at 4 ℃. Then, the 4T1 cells were frozen and thawed in liquid nitrogen for 6 cycles and disrupted by sonication in an ice bath for 20 min. Finally, the large organelle was removed by centrifugation (460 x g, 10 min), and the supernatant was collected to obtain TCL.

The DCs were isolated from the femoral and tibial bones of Balb/c mice using the method previously reported [3]. In brief, the mouse bone marrow cells were collected and cultured with RPMI-1640 medium containing 10% fetal bovine serum, 1% penicillin-streptomycin, and 20 ng/mL GM-CSF for 5 days to obtain immature DCs. Next, the immature DCs were co-incubated with TCL for 24 h to obtain activated mature DCs. The maturity rate of these DCs was evaluated by measuring the expression level of CD80, CD86, and CD40 using a flow cytometer (CytoFLEX, Beckman Coulter, USA). To acquire the TCL-activated mature DC membrane, the mature DCs were collected and lysed with hypotonic lysis buffer containing protease inhibitor cocktail for 1 h at 4 ℃, followed by centrifuging at 4 ℃ (700 x g, 10 min). The resultant supernatant was further centrifuged at 14000 x g for 30 min at 4 ℃. Then, the obtained precipitate was the TCL-activated mature DC membranes.

To construct the IIN@M, the IIN and TCL-activated mature DC membrane were mixed at a weight ratio of 1:1, followed by sonication in an ice bath for 5 min. Then, the mixture was physically extruded using polycarbonate films with pore sizes of 800 and 450 nm at least 10 times. The presence of TCL-activated mature DC membrane in IIN@M was detected using Sodium dodecyl sulphate-polyacrylamide gel electrophoresis (SDS-PAGE).

The percentage of IIN successfully coated with TCL-activated mature DC membrane was evaluated using the DiO probe. Briefly, the DiO solution was mixed with IIN and IIN@M solution (30 *μ*g/mL), respectively. The final concentration of DiO was 2 *μ*M. Then, they were incubated at 37 ℃ for 30 min. Finally, the fluorescence intensity was detected using by a flow cytometer (CytoFLEX, Beckman Coulter, USA).

**1.5** ***In vitro* drug release**

*In vitro* drug release of ICG, IIN, and IIN@M was evaluated using a dialysis method. Briefly, 1 mL of ICG, IIN, and IIN@M solution was added into dialysis bags (3500 Da) and immersed in 40 mL of PBS (pH 7.4). At predetermined time points (2, 4, 8, 12, 24, 48, and 72 h), 1 mL of the solution outside the dialysis bag was withdrawn, followed by the addition of 1 mL of fresh PBS. The cumulative drug release of ICG was then calculated based on the volume of liquid collected at each time point.

**1.6 Detection of multienzyme-like activity**

**1.6.1 Extracellular •OH generation**

The IIN or IIN@M was added to the TMB (5 *μ*g/mL) solution (pH 6.5) with or without H_2_O_2_ (50 *μ*M). The concentration of IIN and IIN@M was both 100 *μ*g/mL. After reaction for 20 min, the UV-Vis spectra of solutions from 500-750 nm were recorded using a spectrophotometer (UV2355, Unico, China). The group of H_2_O_2_ was carried out as a control. To determine the Michaelis-Menten Kinetic Parameters of IIN@M, different concentrations of H₂O₂ (6.25, 12.5, 25, 50, 100 mM) were prepared. 30 *μ*g/mL of IIN@M was added to H_2_O_2_ solution (pH = 6.5) contain TMB (100 mM). The reaction was allowed to proceed for 5 min. The kinetic parameters were calculated according to the Michaelis-Menten equation.

**1.6.2 Detection of extracellular GSH depletion**

The IIN or IIN@M was added to the GSH (10 mM) solution, and the concentration of IIN and IIN@M was both 100 *μ*g/mL. After reaction for 12 h, the mixture was centrifuged (21100 x g) for 10 min. Then, the supernatant was mixed with the DTNB (10 mM) solution. After reaction for 30 min, the UV-Vis spectra of solutions from 350-500 nm were recorded using a spectrophotometer (UV2355, Unico, China). The group of GSH and GSH + H_2_O_2_ served as negative and positive controls, respectively.

**1.6.3 Detection of extracellular O_2_ generation**

There are three groups: H_2_O_2_, H_2_O_2_+IIN (100 *μ*g/mL), and H_2_O_2_+IIN@M (100 *μ*g/mL). The concentration of H_2_O_2_ was 1 mM. The concentration of O_2_ was monitored by a portable dissolved oxygen meter (JPBJ-608, Rex, China) in real time.

**1.7 Measurement of photothermal performance**

The concentration-dependent photothermal performance of IIN@M was measured by exposing 1 mL of IIN@M solution with different concentrations (5, 10, 20, and 40 *μ*g/mL) to an 808 nm NIR laser (EB21712, Changchun New Industries Optoelectronics Tech Co., Ltd., China) at a power density of 1 W/cm^2^. The power density-dependent (0, 0.5, 1.0, 1.5, and 2.0 W/cm^2^) photothermal performance of IIN@M was measured by exposing 1 mL of IIN@M solution (20 *μ*g/mL) to the 808 nm NIR laser. The temperature was measured by an IR thermal camera (Ti200, Fluke, USA). To evaluate the photothermal stability, 1 mL of ICG (20 *μ*g/mL) and IIN@M (20 *μ*g/mL) solution was respectively exposed to the 808 nm NIR laser (0.75 W/cm^2^, 4 min) followed by naturally cooling to room temperature for 4 cycles. According to the previously described Equation, the photothermal conversion efficiency (η) of IIN@M was calculated as follows:

$\eta=\frac{hA\triangle T_{max}-Q_{s}}{I(1-{10}^{-A})}$ Equation (1)

where *h* is the coefficient of heat transfer, A is the container surface area, *ΔT_max_* is the temperature change of the IIN@M solution, *I* is the power density of the NIR laser, *A* is the absorbance of the solution of IIN@M at 896 nm, and *Qs* is the heat associated with the light absorbance of the solution.

DPBF was used to specifically evaluate ^1^O_2_ generation. The groups included control, ICG + L, IIN + L, and IIN@M + L. The concentration of ICG was 10 *μ*g/mL, and that of DPBF was 10 *μ*g/mL. The laser groups were all irradiated using an 808 nm laser (1 W/cm^2^) for 5 min, followed by detection by a UV spectrophotometer (UV2355, Unico, China).

**1.8 Cellular uptake**

4T1 cells were seeded in 12-well plates at a density of 1 x 10^5^ cells per well and incubated overnight. Next, the old medium was removed. The cells were incubated in fresh medium containing ICG, IIN, or IIN@M for 2, 4, and 8 h. Subsequently, the cells were collected, and the cellular uptake was analyzed by the flow cytometer (CytoFLEX, Beckman Coulter, USA). Additionally, the cells were stained with Hoechst 33342 for 15 min and observed by Confocal Laser Scanning Microscopy (CLSM, AIR HD25, Nikon, Japan).

**1.9 Cell viability assay**

The cell viability was investigated by MTT assay. Briefly, 4T1 cells were seeded in 96-well plates at a density of 1 x 10^4^ cells per well. After adhering overnight, the medium was discarded, and cells were incubated in 100 *μ*L of fresh medium containing various concentrations of ICG, IIN, or IIN@M (5, 10, 20, 30, and 40 *μ*g/mL). after incubation for 8 h, the groups of ICG + L, IIN + L, and IIN@M + L were exposed to the 808 nm laser (1 W/cm^2^, 5 min) for photothermal therapy (PTT) *in vitro*, followed by continuous incubation for another 12 h. Then, the MTT was added and continuously incubated for 4 h. Afterward, the supernatants were removed, and 100 *μ*L DMSO was added to the 96-well plates. Finally, the absorbance at 490 nm of cells was recorded by a microplate reader (SpectraMax Id5, Molecular Devices, USA), and the cell viability was calculated.

The cell viability of normal HEK293 cells was evaluated by similar methods. HEK293 cells were seeded in 96-well plates at a density of 1 x 10^4^ cells per well. After adhering overnight, the medium was discarded, and cells were incubated in 100 *μ*L of fresh medium containing various concentrations of ICG, IIN, or IIN@M (5, 10, 20, 30, and 40 *μ*g/mL). Then, the MTT assay was conducted as mentioned above.

**1.10 Cell Apoptosis assay**

4T1 cells were seeded in 12-well plates at a density of 1 x 10^5^ cells per well and incubated overnight. After different treatments (Control, ICG, IIN, IIN@M, ICG+L, IIN+L, IIN@M+L), the cells were incubated for another 12 h. 808 nm laser treatment was carried out for 5 min under the condition of 1 W/cm^2^. Then, the cells were treated according to the procedure of the Annexin V-FITC Apoptosis Detection Kit (Beyotime, China) and detected by the flow cytometer (CytoFLEX, Beckman Coulter, USA).

**1.11 Detection of intracellular ROS generation**

4T1 cells were seeded in 12-well plates at a density of 1 x 10^5^ cells per well and incubated overnight. Afterward, the cells were incubated in medium with different formulations (Control, ICG, IIN, IIN@M, ICG+L, IIN+L, IIN@M+L) for 8 h. After 808 nm laser (1 W/cm^2^, 5 min) irradiation, the cells were incubated with DCFH-DA probe for 20 min at 37 ℃. Then, the intracellular ROS was measured by the flow cytometer (CytoFLEX, Beckman Coulter, USA) and CLSM (AIR HD25, Nikon, Japan).

For ^1^O_2_ generation, the 4T1 cells with different treatments were incubated with the SOSG probe for 20 min at 37 ℃. Then, the intracellular ^1^O_2_ was measured by the flow cytometer (CytoFLEX, Beckman Coulter, USA).

**1.12 Detection of intracellular GSH, Dihydronicotinamide adenine dinucleotide phosphate (NADPH), and Lipid Peroxidation (LPO)**

4T1 cells were seeded in 12-well plates at a density of 2 x 10^5^ cells per well and incubated overnight. After different treatments (Control, ICG, IIN, IIN@M, ICG+L, IIN+L, IIN@M+L), the cells were incubated for another 12 h. 808 nm laser treatment was carried out for 5 min under the condition of 1 W/cm^2^. Then, the cells were collected for GSH and NADPH assay using GSH Assay Kit (Beyotime, China) and NADPH Assay Kit (Beyotime, China) following the manual protocols, respectively. For measuring the intracellular LPO level, the collected cells were incubated with BODIPY^581/591^ C11 probe at 37 ℃ for 20 min. Afterward, intracellular LPO level was measured using the flow cytometer (CytoFLEX, Beckman Coulter, USA) and observed using CLSM (AIR HD25, Nikon, Japan).

**1.13 Detection of intracellular O_2_ generation**

4T1 cells were implanted into a glass-bottom confocal dish and cultivated for 24 h. Then, the old medium was replaced with the fresh medium containing ICG, IIN, and IIN@M, followed by incubation for 12 h. Subsequently, the [Ru(dpp)_3_] Cl_2_ (20 *μ*M) probe was added and incubated for another 1 h. Then, the cells were washed using PBS and observed using CLSM (AIR HD25, Nikon, Japan).

**1.14 Detection of ATP, HMGB1, and CRT**

4T1 cells were seeded in 6-well plates at a density of 2 x 10^5^ cells per well. After attachment overnight, the cells were incubated in medium with different formulations (Control, ICG, IIN, IIN@M, ICG+L, IIN+L, IIN@M+L) for 12 h. 808 nm laser treatment was performed for 5 min under the condition of 1 W/cm^2^. The supernatant was collected to assay the release of ATP using the ATP Assay Kit (Beyotime, China). The intracellular level of HMGB1 and CRT was observed using CLSM (AIR HD25, Nikon, Japan), after incubating with FITC-labelled anti-HMGB1 and CRT antibodies, respectively.

**1.15 Evaluation of mitochondrial function**

4T1 cells were seeded in 6-well plates at a density of 2 x 10^5^ cells per well. After attachment overnight, the cells were incubated in medium with different formulations (Control, ICG, IIN, IIN@M, ICG+L, IIN+L, IIN@M+L) for 12 h. 808 nm laser treatment was performed for 5 min under the condition of 1 W/cm^2^. To observe the mitochondrial ROS production, the cells were incubated with MitoSOX Red probe at 37 ℃ for 30 min. The mitochondrial ROS level was observed by CLSM (AIR HD25, Nikon, Japan). Additionally, the cells were incubated with JC-1 probe to evaluate mitochondrial membrane potential by the flow cytometer (CytoFLEX, Beckman Coulter, USA). Finally, the cells were treated following the manual protocols of the mitochondrial permeability transition pore (mPTP) assay kit (Abbkine, China) to investigate the opening of mitochondrial mPTP.

**1.16 Detection of DNA oxidative damage**

4T1 cells were seeded in a glass-bottom confocal dish at a density of 1 x 10^5^ cells per well. After attachment overnight, the cells were incubated in medium with different formulations (Control, ICG, IIN, IIN@M, ICG+L, IIN+L, IIN@M+L) for 12 h. 808 nm laser treatment was performed for 5 min under the condition of 1 W/cm^2^. Then, the cells were incubated with Mito tracker and anti-8-OHdG antibody at 37 37 ℃ for 30 min. Afterward, cells were incubated with FITC-labeled second antibody and stained with Hoechst 33342. Finally, the mitochondrial oxidative DNA damage was measured using CLSM (AIR HD25, Nikon, Japan).

**1.17 Detection of cytosol mitochondrial DNA (mtDNA)**

Cytosol mtDNA released from mitochondria was measured using quantitative polymerase chain reaction (qPCR) as previously reported in the literature with minor modifications [4]. In brief, 4T1 cells were seeded in a 6-well plate at a density of 2 x 10^5^ cells per well. After attachment overnight, the cells were incubated in medium with different formulations (Control, ICG, IIN, IIN@M, ICG+L, IIN+L, IIN@M+L) for 12 h. 808 nm laser treatment was performed for 5 min under the condition of 1 W/cm^2^. Subsequently, the cells were treated with mitochondria isolation buffer on ice ​for 10 min and homogenized by the glass homogenizer for 20 strokes. The cell homogenate was centrifuged at 600 × g for 10 min at 4°C to remove unbroken cells and nuclei. The supernatant was further centrifuged at 11,000g × g for 10 min at 4°C to precipitate mitochondria, and the resultant supernatant was regarded as the cytosolic fraction. The cytosolic DNA was extracted using a genomic DNA extraction kit (Beyotime, China). qPCR was performed on cytosolic DNA using mtDNA (ND1) primers (provided in **Table S1**).

**1.18 Western bolt analysis**

4T1 cells were seeded in 6-well plates at a density of 2 x 10^5^ cells per well. After attachment overnight, the cells were incubated in medium with different formulations (Control, ICG, IIN, IIN@M, ICG+L, IIN+L, IIN@M+L) for 12 h. 808 nm laser treatment was performed for 5 min under the condition of 1 W/cm^2^. Next, the cells were harvested and lysed using radioimmunoprecipitation assay (RIPA) buffer with inhibitors of proteases and phosphatases. The concentration of protein was measured by the BCA Quantitation Kit (Servicebio, China) to ensure equal amounts for further research. The obtained protein samples were separated by SDS-PAGE and transferred to polyvinylidene difluoride (PVDF) membrane (Millipore, Germany). The PVDF membrane was blocked in 5% skim milk powder solution (w/v) for 2 h, subsequently incubated overnight at 4 ℃ with rabbit-derived primary antibody against *β*-actin, cleaved caspase 3, GSDME, GPX4, HIF-1*α*, SLC7A11, SLC1A1, IDO1, cGAS, p-STING, p-TBK1, and p-IRF3. Finally, the PVDF membrane was incubated with HRP-labeled second antibodies for 1 h and visualized using an enhanced chemiluminescence kit (Biosharp, China) by gel imaging system (Chemi Doc MP, Bio-Rad, USA).

**1.19 Detection of cytokine generation**

4T1 cells were seeded in 6-well plates at a density of 2 x 10^5^ cells per well. After attachment overnight, the cells were incubated in medium with different formulations (Control, ICG, IIN, IIN@M, ICG+L, IIN+L, IIN@M+L) for 12 h. 808 nm laser treatment was performed for 5 min under the condition of 1 W/cm^2^. The supernatant was collected to measure the level of IFN-*β*, TNF-*α*, and IL-6 according to the protocol of the corresponding ELISA Kit (Elabscience, C).

**1.20 Detection of the activation of DC and polarization of macrophage *in vitro***

Murine DCs were extracted and cultured according to the method mentioned in section 1.4. Then, the DCs were co-cultured with the supernatant obtained from 4T1 with different treatments (Control, ICG, IIN, IIN@M, ICG+L, IIN+L, IIN@M+L) for 12 h. Then, the expression of CD86 and CD80 was detected by a flow cytometer (CytoFLEX, Beckman Coulter, USA). To detect the polarization of macrophages i*n vitro*, the RAW 264.7 cells were co-cultured with the supernatant obtained from 4T1 with different treatments (Control, ICG, IIN, IIN@M, ICG+L, IIN+L, IIN@M+L) for 12 h. Then, the expression of CD86 and CD206 was detected by flow cytometer (CytoFLEX, Beckman Coulter, USA).

**1.21 Detection of IDO enzyme activity**

The IDO enzyme activity assay was performed according to the methods reported previously [5]. In detail, 4T1 cells were seeded in 12-well plates at a density of 1 x 10^5^ cells per well. After attachment overnight, the cells were incubated in medium with different formulations (Control, ICG, IIN, IIN@M, ICG+L, IIN+L, IIN@M+L) for 12 h. 808 nm laser treatment was performed for 5 min under the condition of 1 W/cm^2^. The supernatant (100 *μ*L) was mixed with 10 *μ*L of 30% (w/v) trichloroacetic acid solution and incubated at 50 ℃ for 30 min. Then, the mixture was centrifuged at 3000g for 10 min, and the supernatant (100 *μ*L) was mixed with p-dimethylaminobenzaldehyde/acetic acid (100 *μ*L). The absorbance of the mixture at 360 nm was recorded using the microplate reader (SpectraMax Id5, Molecular Devices, USA).

**1.22 Evaluation of antitumor efficiency *in vivo***

Female BALB/c mice (6-8 weeks old) were obtained from BIORN Life Science Co., Ltd. (Nanjing, China). All the animal operations conformed to the guidelines set by the Institutional Animal Care and Use Committee. The primary tumors were established by subcutaneous injection of 4T1 cells (1 x 10^6^). After 4 days, the distant tumors were established by subcutaneous injection of 4T1 cells (1 x 10^6^). After 3 days, mice were randomly divided into 8 groups: Control, ICG, NLG8189, IIN, IIN@M, ICG+L, IIN+L, IIN@M+L (*n* = 5 per group). The drugs were intratumorally injected at the site of primary tumors with a dose of 10 mg/kg. After injection for 8 h, 808 nm laser irradiation was performed in laser groups (1 W/cm^2^, 4min). The growth of the tumor was monitored every 3 days and calculated according to the formula as follows:

$\mathrm{Tu}$mor volume = $\frac{\left( tumor length \right) \times{(tumor width)}^{2}}{2}$ Equation (2)

After treatment for 15 days, the mice were sacrificed, and their tumors were collected for H&E staining and immunofluorescence analysis. The major organs, including the heart, liver, spleen, lung, and kidney, were harvested for H&E staining.

To study the *in vivo* tumor targeting effect, ICG, IIN, and IIN@M were tail vein injected into 4T1-tumor-bearing BALB/c mice. After injection for 8 h, the mice were sacrificed, and their hearts, livers, spleens, lungs, spleens, and tumors were collected for NIR *ex vivo* fluorescence imaging analysis using the IVIS Spectrum optical imaging system (IVIS Spectrum, PerkinElmer, USA).

**1.23 Evaluation of immune response *in vivo***

To evaluate the antitumor immune response *in vivo*, the primary tumor and distant tumor were harvested and prepared to form a single-cell suspension according to the method reported previously [6]. The resultant cell suspension was stained with antibodies against CD3, CD4, and CD8 to analyze T cells. The suspension was stained with antibodies against CD11c, CD80, and CD86 to analyze DCs. The suspension was stained with antibodies against CD11b, F4/80, CD86, and CD206 to analyze macrophages. Then, these cell suspensions were detected by a flow cytometer (CytoFLEX, Beckman Coulter, USA).

**1.24 Statistical Analysis**

Data were analyzed using Graph Pad Prism, and data were expressed as the mean ± SD of at least three repeated measurements. *t*-tests (two-sided) were performed to analyze the data. *p* < 0.05 was considered statistically significant. **p* < 0.05, ***p* < 0.01, and ****p* < 0.001.

**2 Supplementary tables and figures**

**Table S1**. Primers utilized in qPCR.

| **Gene** | **Forward primer sequence** | **Reverse primer sequence** |
| --- | --- | --- |
| 18S | GTAGTCATATGCTTGTCTC | CTTCCGTCAATTCCTTTAAC |
| ND1 | TCCGAGCATCTTATCCACGC | GTATGGTGGTACTCCCGCTG |

**
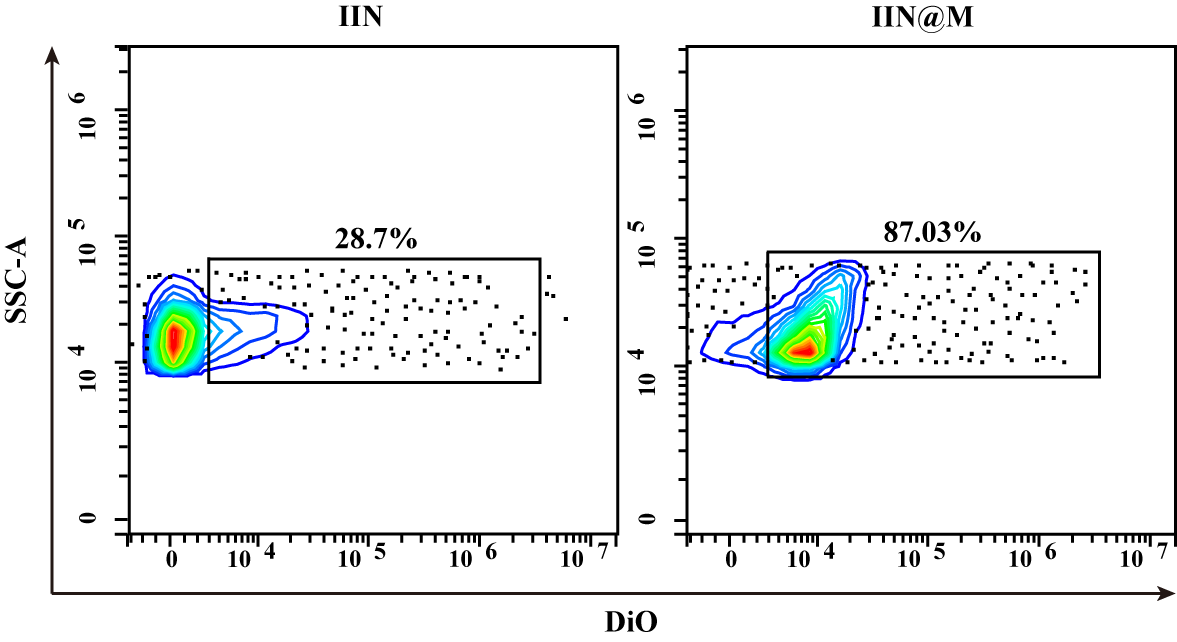
**

**Figure S1.** The percentage of IIN successfully coated with TCL-activated mature DC membrane.


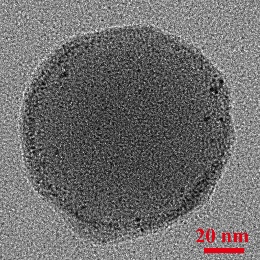


**Figure S2.** TEM image of IIN@M.


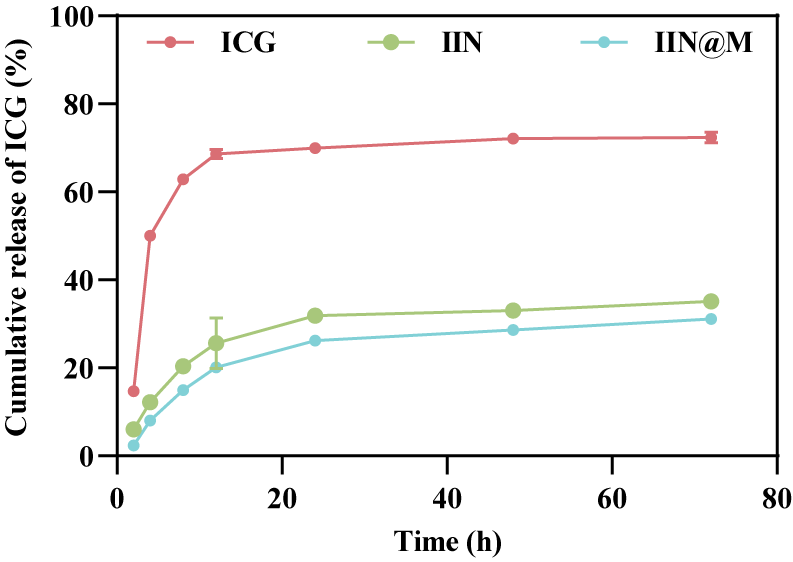


**Figure S3.** Cumulative release curves of ICG, IIN, and IIN@M in PBS. Data are expressed as mean ± SD. *n*=3.

**
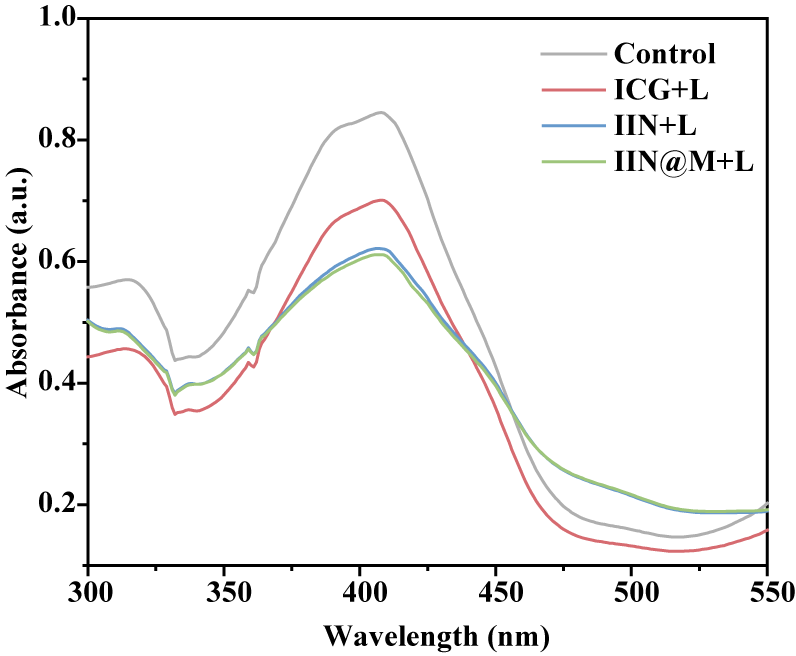
**

**Figure S4.** Determination of ^1^O_2_ production after 808 nm laser radiation (1 W/cm^2^, 5 min).


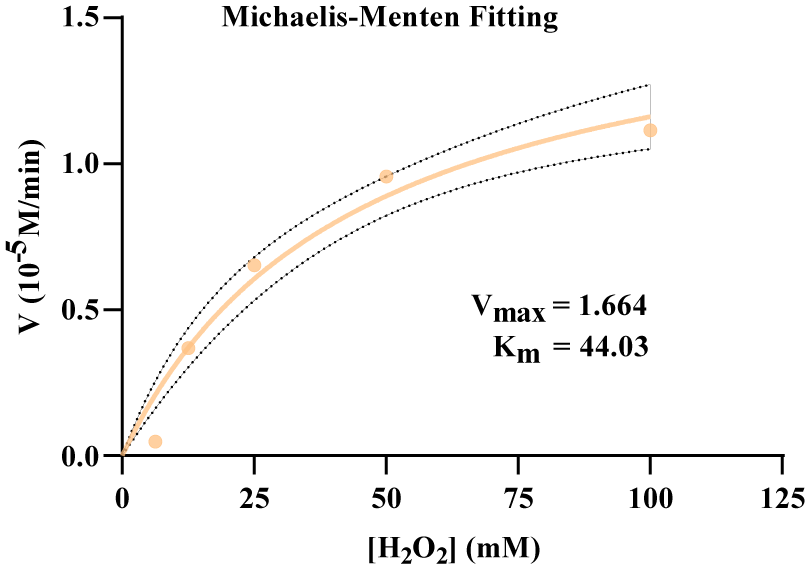


**Figure S5.** Michaelis-Menten Kinetic Parameters of POD-like activity of IIN@M.


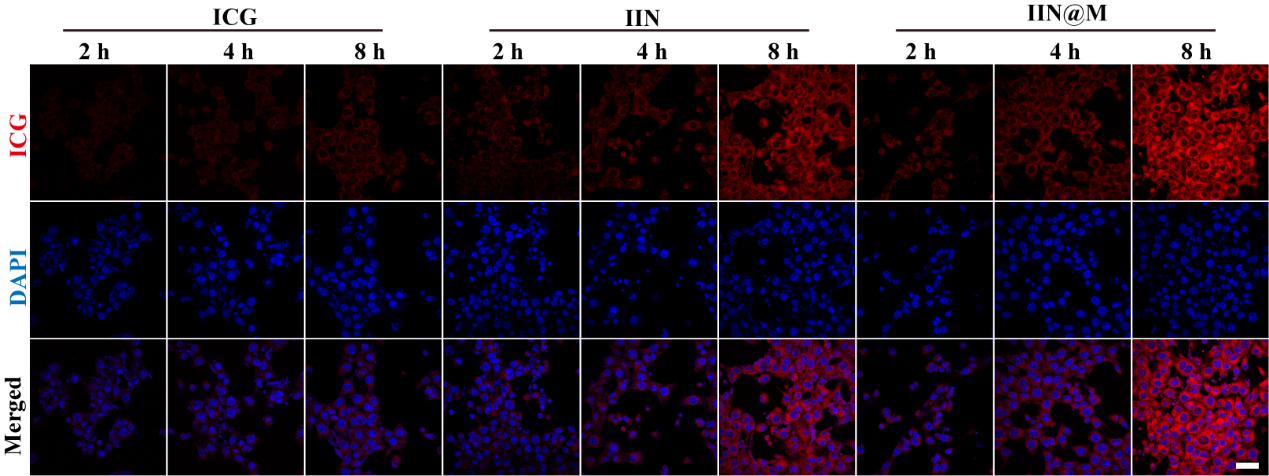


**Figure S6.** CLSM analysis of cell uptake by 4T1 cells after incubation with ICG, IIN, and IIN@M for 2, 4, and 8 h (scale bar = 50 *μ*m).


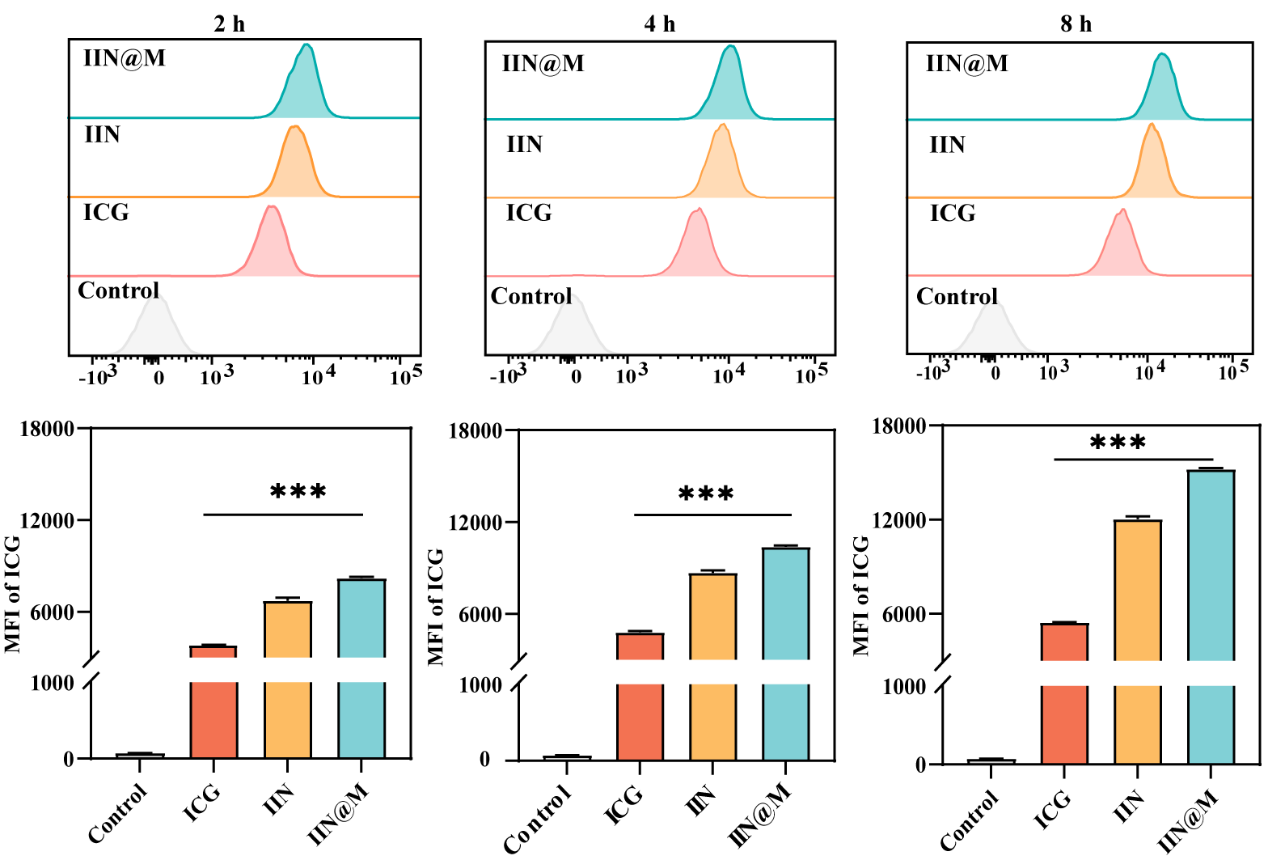


**Figure S7.** Flow cytometric analysis of cell uptake by 4T1 cells after incubation with ICG, IIN, and IIN@M for 2, 4, and 8 h. Data are expressed as mean ± SD (*n* =3). * *p* < 0.05; ** *p* < 0.01; *** *p* < 0.001.


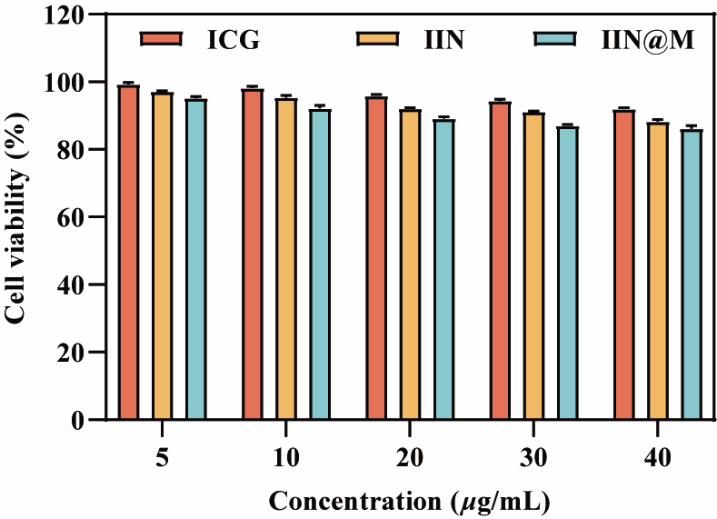


**Figure S8.** Cell viability of HEK293 cells after different treatments. Data are expressed as mean ± SD (*n* = 6).


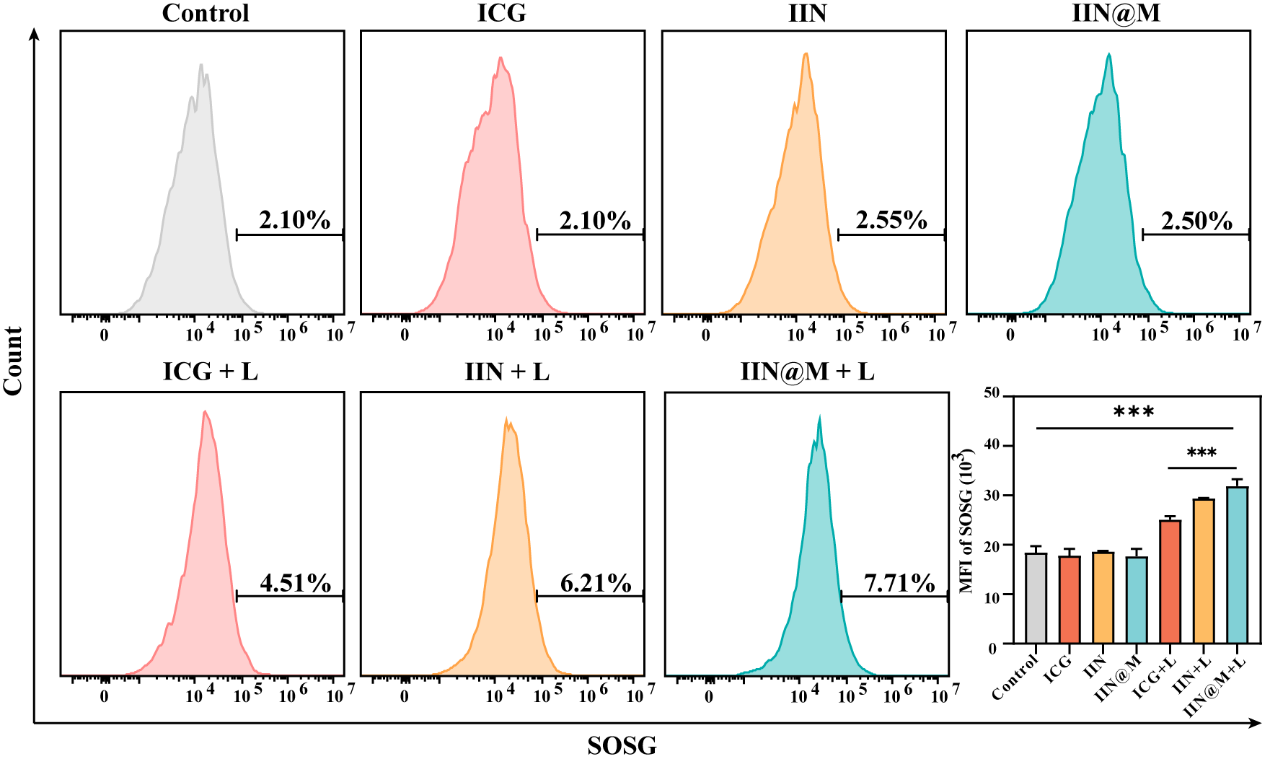


**Figure S9.** Intracellular level of ^1^O_2_ after different treatments. Data are expressed as mean ± SD (*n* =3). * *p* < 0.05; ** *p* < 0.01; *** *p* < 0.001.


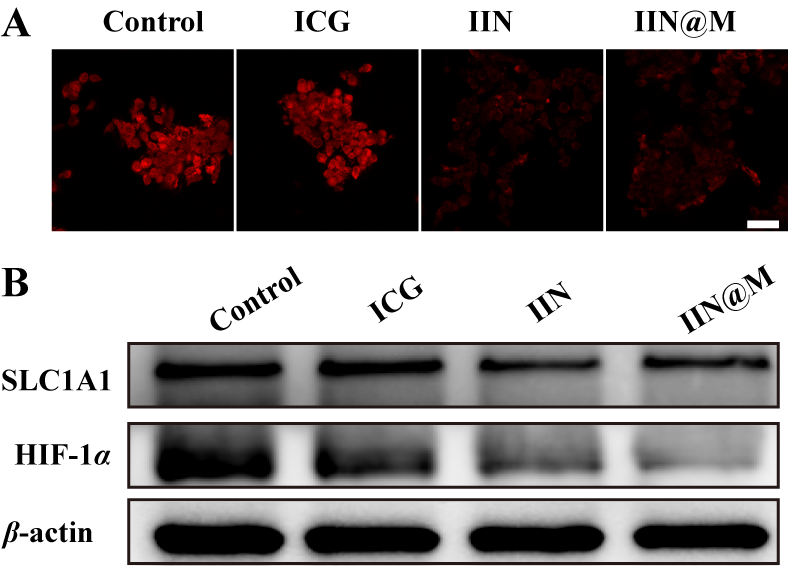


**Figure S10.** Intracellular O_2_ level (A) and the expression level of SLC1A1 and HIF-1*α* (B) in 4T1 cells with different treatments (scale bar = 50 *μ*m).


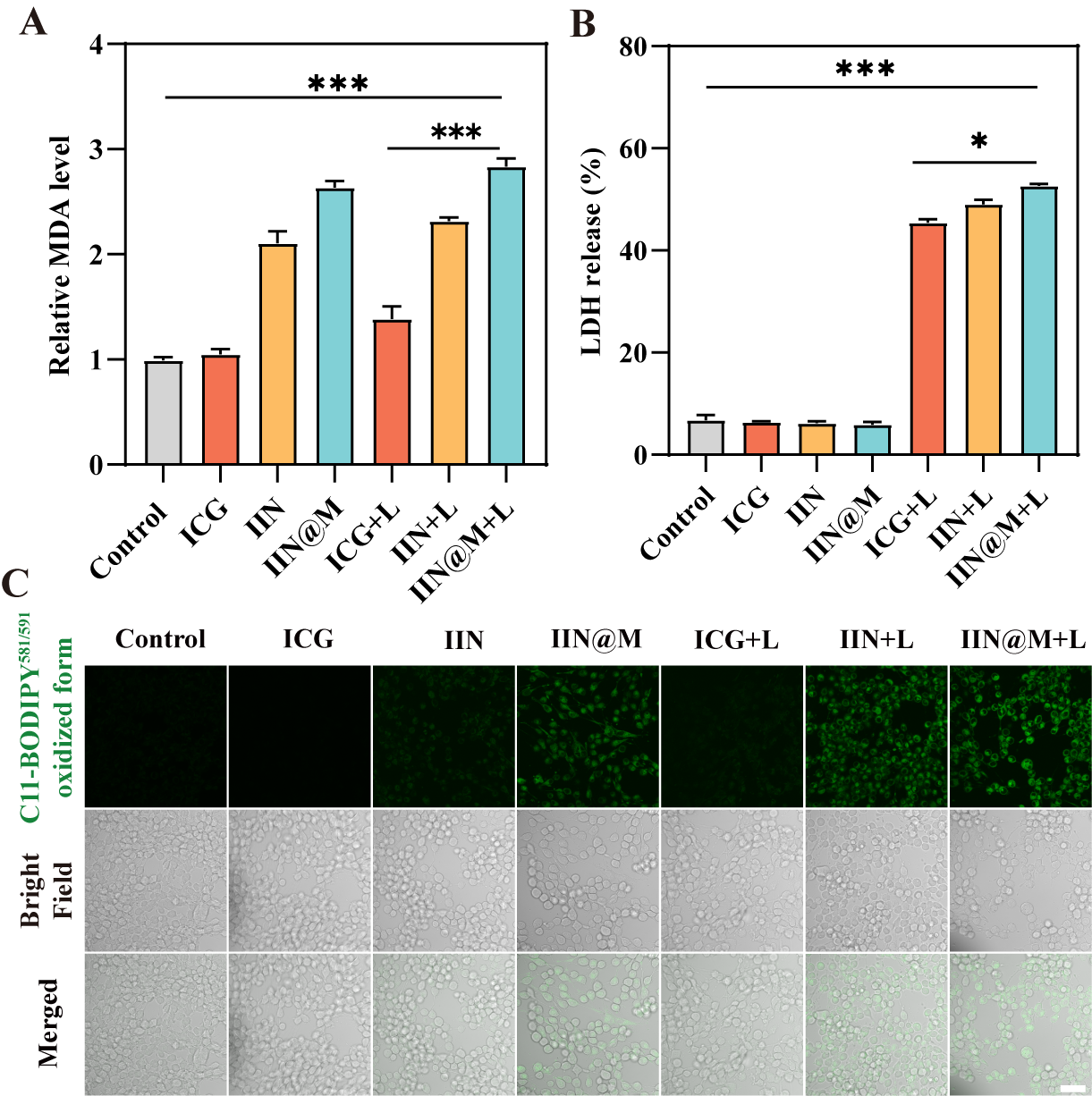


**Figure S11.** The LDH release (A), intracellular MDA (B), and LPO (C) levels of 4T1 cells after different treatments (scale bar = 50 *μ*m). Data are expressed as mean ± SD (*n* =3). * *p* < 0.05; ** *p* < 0.01; *** *p* < 0.001.


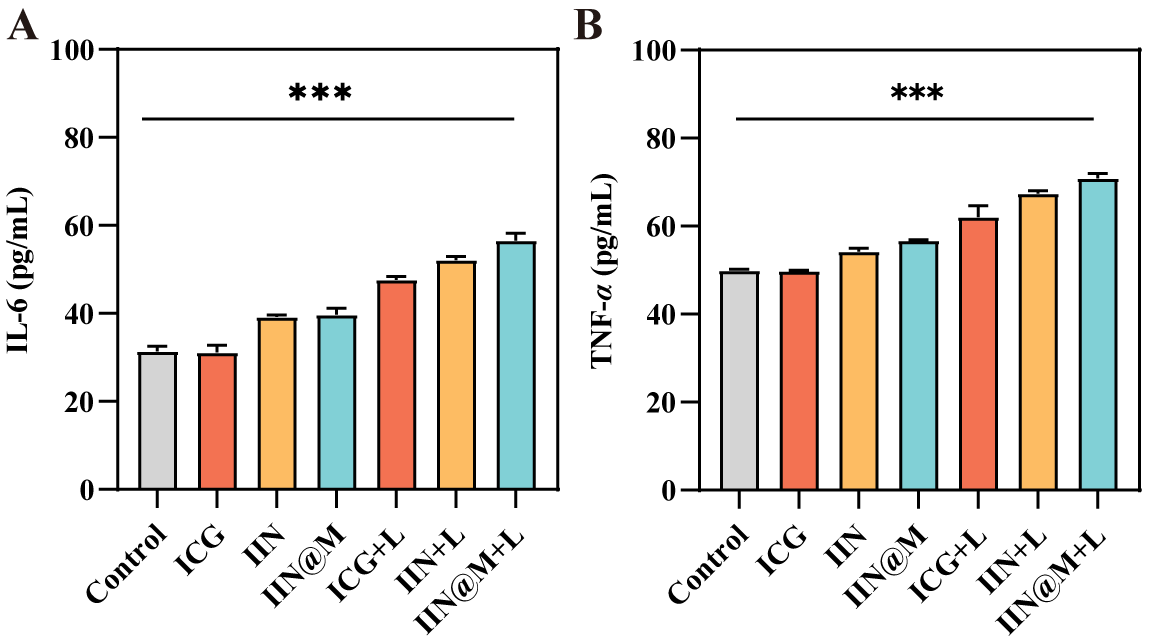


**Figure S12.** ELISA measurement of IL-6 (A) and TNF-*α* (B) in cocultivation suspension of 4T1 cells with different treatments. Data are expressed as mean ± SD (*n* =3). * *p* < 0.05; ** *p* < 0.01; *** *p* < 0.001.


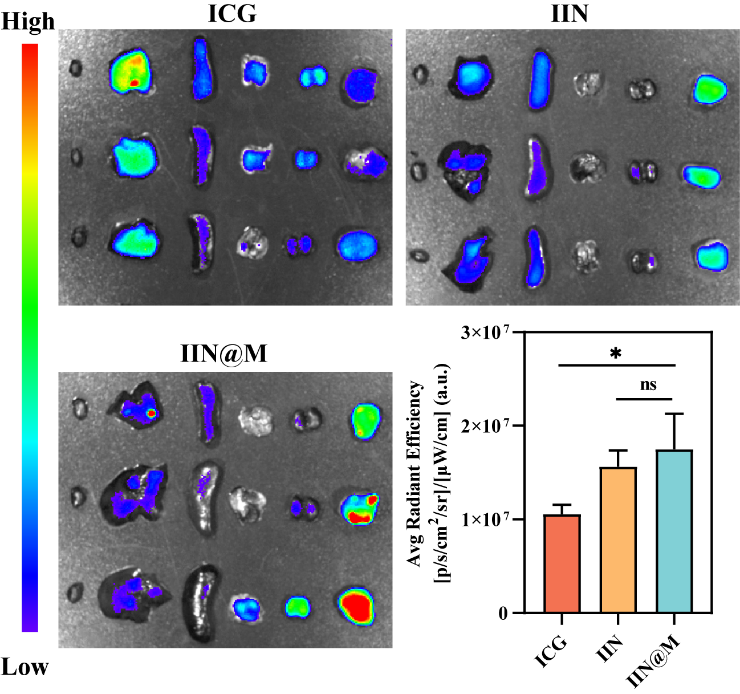


**Figure S13.** *Ex vivo* fluorescence imaging of ICG, IIN, and IIN@M in major tissues (heart, liver, spleen, lung, and kidney) and tumors after intravenous injection for 8 h. Left to right is heart, liver, spleen, lung, kidney, and tumor, respectively. Data are expressed as mean ± SD. *n*=3. **p* < 0.05;


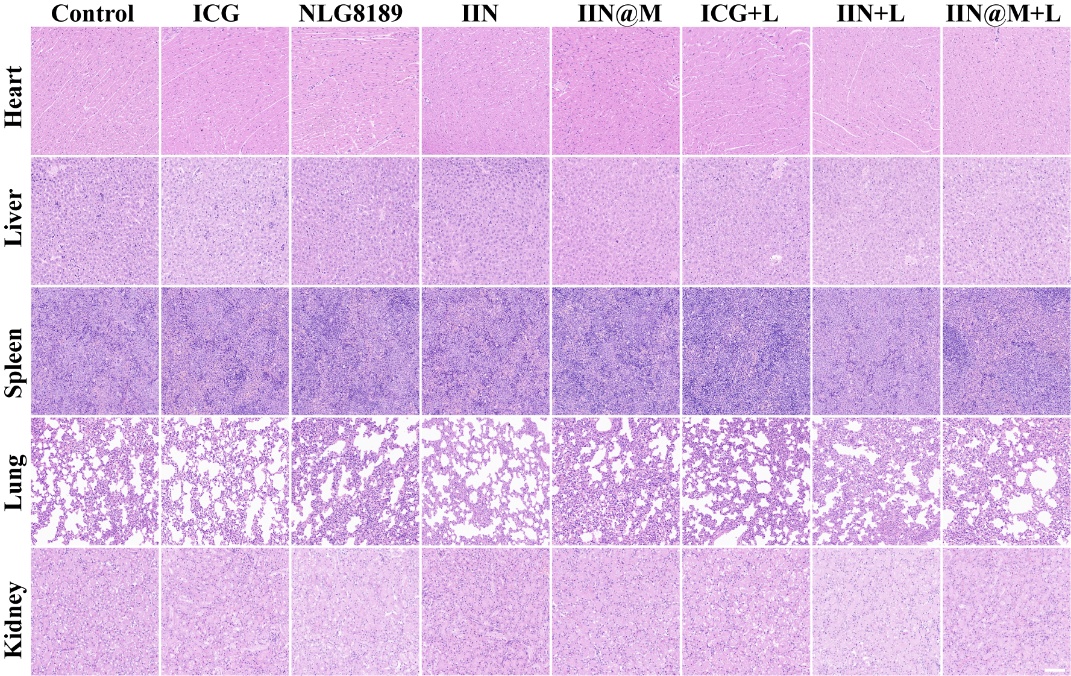


**Figure S14.** H&E staining images of major organs (heart, liver, spleen, lung, and kidney), scale bar = 100 *μ*m.


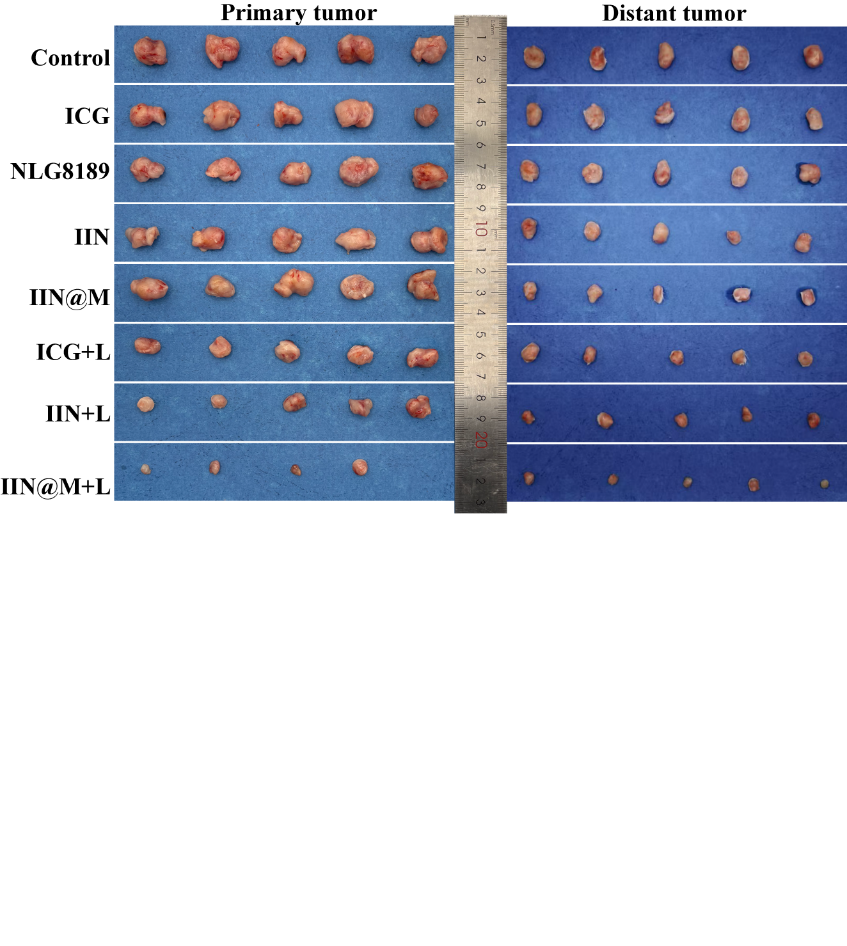


**Figure S15.** Optical photograph of primary and distant tumor tissue.

**Reference**

1. Cao YM, Long JR, Sun HS, Miao YQ, Sang Y, Lu HT, Yu CX, Zhang Z, Wang L, Yang J, Wang SQ: Dendritic Cell-Mimicking Nanoparticles Promote mRNA Delivery to Lymphoid Organs. Advanced Science 2023, 10.

2. Ma XY, Kuang L, Yin Y, Tang L, Zhang Y, Fan Q, Wang BY, Dong ZF, Wang W, Yin TY, Wang YZ: Tumor-Antigen Activated Dendritic Cell Membrane-Coated Biomimetic Nanoparticles with Orchestrating Immune Responses Promote Therapeutic Efficacy against Glioma. Acs Nano 2023, 17:2341-2355.

3. Liu WL, Zou MZ, Liu T, Zeng JY, Li X, Yu WY, Li CX, Ye JJ, Song W, Feng J, Zhang XZ: Cytomembrane nanovaccines show therapeutic effects by mimicking tumor cells and antigen presenting cells. Nature Communications 2019, 10.

4. Zhu XQ, Wang XX, Liu ZM, Jiang B, He ZH, Liu SJ, Wu YH, Wu ZX, Zhang TT, Liu MY, et al: Peroxidase-Like Nanozyme Activates the cGAS-STING Pathway via ROS-Induced mtDNA Release for Cancer Immunotherapy. Advanced Functional Materials 2024, 34.

5. Li Q, Zhang D, Zhang J, Jiang Y, Song AX, Li ZH, Luan YX: A Three-in-One Immunotherapy Nanoweapon via Cascade-Amplifying Cancer-Immunity Cycle against Tumor Metastasis, Relapse, and Postsurgical Regrowth. Nano Letters 2019, 19:6647-6657.

6. Feng QS, Qi FG, Fang WM, Hu P, Shi JL: Ferroptosis to Pyroptosis Regulation by Iron-Based Nanocatalysts for Enhanced Tumor Immunotherapy. Journal of the American Chemical Society 2024, 146:32403-32414.
